# Biostimulant activity of *Galaxaura rugosa* seaweed extracts against water deficit stress in tomato seedlings involves activation of ABA signaling

**DOI:** 10.3389/fpls.2023.1251442

**Published:** 2023-09-14

**Authors:** Sarai Morales-Sierra, Juan Cristo Luis, David Jiménez-Arias, Nereida M. Rancel-Rodríguez, Alberto Coego, Pedro L. Rodriguez, Mercedes Cueto, Andrés A. Borges

**Affiliations:** ^1^ Grupo de Biología Vegetal Aplicada (GBVA), Departamento de Botánica, Ecología y Fisiología Vegetal, Facultad de Farmacia Universidad de La Laguna, La Laguna, Tenerife, Spain; ^2^ Departamento de Producción Vegetal en Zonas Tropicales y Subtropicales, Instituto Canario de Investigaciones Agrarias (ICIA), La Laguna, Tenerife, Spain; ^3^ Grupo BotMar-ULL, Departamento de Botánica, Ecología y Fisiología Vegetal, Facultad de Farmacia Universidad de La Laguna, La Laguna, Tenerife, Spain; ^4^ Instituto de Biología Molecular y Celular de Plantas, Consejo Superior de Investigaciones Científicas, Universidad Politécnica de Valencia, Valencia, Spain; ^5^ Departamento de Ciencias de la Vida y de la Tierra, Departamento de Productos Naturales y Sintéticos Bioactivos, Instituto de Productos Naturales y Agrobiología (IPNA-CSIC), La Laguna, Tenerife, Spain

**Keywords:** biostimulants for agriculture, water deficit, drought, seaweed, bioactive natural products, absicic acid signaling

## Abstract

Water scarcity is a serious constraint for agriculture, and global warming and climate change can exacerbate it in many areas. Therefore, sustainable approaches must be implemented to deal with current and future water scarcity scenarios. Genetic and chemical approaches are being applied to manage this limitation and maintain crop yields. In particular, biostimulants obtained from natural sources such as marine algae are promising aids for coping with water deficit stress in agriculture. Here we present a bioprospection study of extracts of the macroalgae *Bonnemaisonia hamifera*, *Galaxaura rugosa*, *Dasycladus vermicularis*, *Ulva clathrata*, *Cystoseira foeniculacea*, *Cystoseira humilis*, *Lobophora dagamae*, *Colpomenia sinuosa* and *Halopteris scoparia* from the north coast of Tenerife, in the Canary Islands. The aqueous extracts of *Bonnemaisonia hamifera*, *Galaxaura rugosa*, *Dasycladus vermicularis and Cystoseira humilis* show biostimulant activity against water deficit stress in tomato seedlings under controlled conditions, providing higher tolerance than the mock-treated control. The *Galaxaura rugosa* extract showed the highest biostimulant activity against water deficit stress. We demonstrate that this positive effect involves the activation of the abscisic acid (ABA) pathway in *Arabidopsis thaliana* (arabidopsis) and *Solanum lycopersicum* (tomato). Application of *G. rugosa* extract to the root system by drenching tomato seedlings subjected to water deficit leads to improved CO_2_ assimilation and water use efficiency (WUEp), compared to mock-treated plants. These results highlight a new potential seaweed source of substances with osmoprotectant properties, useful for biostimulant development. Future studies may provide further insight into which components of the seaweed extract induce activation of the ABA pathway.

## 1 Introduction

In recent decades, the exponential development of innovative new technologies has led to the provision of innovative methods for the isolation and identification of natural products. One of the areas of interest is the development of therapeutic bioactive compounds from the marine ecosystem (Molinski et al., 2009). The world’s oceans and seas are home to a wide variety of organisms that have evolved complex metabolic capabilities to adapt to their habitat and produce a range of secondary metabolites with useful biological activities. Among them, marine biotechnology is an emerging field based on the exploration and exploitation of marine resources (de Vera et al., 2018).

One application of marine natural products is the use of seaweeds and microalgae as biostimulants for agriculture (Bulgari et al., 2019; Mazepa et al., 2021). In recent years, due to population growth levels that could seriously threaten food security, the agricultural industry has expanded the search for novel compounds with an environmentally friendly profile. This is in line with the “Green Deal” and “Farm to Fork” strategies adopted by EU policies, being driven by global awareness of the need to develop sustainable production systems (Mazepa et al., 2021). For example, this worldwide trend in agricultural markets has increased the extent of certified organic farming, to reach 71.1 million ha, almost doubling the 35.9 million ha in 2013 (Bulgari et al., 2019).

According to the EU Regulation, biostimulants are products that “stimulate plant nutritional processes, irrespective of the nutrient content of the product, with the sole aim of improving one or more of the following characteristics of the plant or its rhizosphere: (a) nutrient use efficiency; (b) tolerance to abiotic stress; (c) quality traits; or (d) availability of limited nutrients in the soil or rhizosphere” According to Du Jardin (du Jardin, 2015), there are seven categories of biostimulants: humic/fulvic acids, seaweed/botanical extracts, protein hydrolysates, biopolymers, beneficial minerals, beneficial bacteria, and beneficial fungi. Another classification by Bulgari et al., 2019 added a new category including extracts from industrial or food waste, and placed nanomaterials and nanoparticles in the biopolymers category (Jiménez-Arias et al., 2021).

Among seaweeds, the brown seaweed *Ascophyllum nodosum* is the most popular source used in agriculture (Xu and Leskovar, 2015; Yang et al., 2019). There are several seaweed-based liquid fertilizer formulations on the market, such as Kelpak™ (Aremu et al., 2016) and Sealgae™. Preparing their extracts requires using water, acid or alkaline treatments, low temperatures, or heating, or disruption using physical methods (Battacharyya et al., 2015). However, for most documented seaweed extracts, the main chemical components responsible for their biostimulant activity are unknown (Ertani et al., 2018).

Salinity and drought are the most important abiotic stresses (Summary for Policymakers, 2014). Together, they are responsible for up to a 75% reduction in global crop production. Moreover, their impact will increase due to climate change (Summary for Policymakers, 2014). These two stresses exert an osmotic shock on plants by lowering the soil water potential due to limited water availability, leading to water deficit, oxidative stress, and nutrient imbalance. The aim of this study was to evaluate the effects of aqueous extracts obtained from nine seaweeds from the north coast of Tenerife in the Canary Islands, and to determine their chemical composition. Among them, the *Galaxaura rugosa* extract showed the highest activity. We then investigated the possible mechanism underlying this activity and found it to be partly mediated by the activation of the ABA signaling pathway. The defense mechanisms against drought include biophysical, biochemical, cellular and molecular processes integrated in plant stress physiology. The improvement of the root system architecture, leaf structure, osmotic balance, relative water content and stomatal aperture modulation are considered to be the most prominent physiological features for drought resistance in crop plants. Moreover, reactive oxygen scavenging and signaling via calcium and phytohormones such as abscisic acid, salicylic acid, jasmonic acid, auxin, gibberellin, ethylene, brassinosteroids and peptide molecules are crucial mechanisms for coping with drought stress (Iqbal et al., 2022). Among all phytohormones, ABA has a crucial role in coping with drought stress (Mega et al., 2019; Yang et al., 2019; Mao et al., 2022). ABA is a key signaling molecule that mediates plant acclimation to water deficit by reducing transpiration, protecting photosynthesis and triggering other metabolic adjustments, including the induction of stress proteins and osmolytes. Consequently, fine-tuning and modulating ABA responses holds the promise of pre-adapting plants to drought through changes in both short and long-term plant physiology (Yang et al., 2019).

These results highlight a new seaweed source potentially capable of protecting plants against water deficit stress, showing notable osmoprotectant properties. Moreover, we unveil the capability of *G. rugosa* seaweed as a novel biostimulant source. Both these uses make it or its constituents a promising resource for commercialization.

## 2 Materials and methods

### 2.1 Algae collection and extraction processes

The nine macroalgae tested in this study were collected along different seasons between 2021 and 2022 at Punta del Hidalgo (28° 33’ 37.32’’ N, 16° 20’ 7.843’’ W) on the north coast of the island of Tenerife (Canary Islands) at low tide in the intertidal zone, in accordance with the Nagoya Protocol, permit reference: ESNC102. These algae were selected for their extensive study in the archipelago in terms of morphology and genetics. Specimen identification was confirmed using a Leica DM 500 Microsystems optical microscope (Wetzlar, Germany), following the classification proposed by Guiry and Guiry in 2023. A voucher specimen of each species was deposited at the University of La Laguna (Tenerife, Spain), see **Table 1**.

**Table 1:** Details of species, biomass, extracts and voucher specimen codes.

Species	Phylum	Dry biomass (g)	Extract (g)	Voucher specimen
*Bonnemaisonia hamifera*	Rhodophyta	49.70	4.52	TFC-Phyc 16441
*Cystoseira foeniculacea*	Ochrophyta	48.43	15.68	TFC-Phyc 16446
*Dasycladus vermicularis*	Chlorophyta	55.00	11.31	TFC-Phyc 16444
*Cystoseira humilis*	Ochrophyta	47.20	12.63	TFC-Phyc 16445
*Galaxaura rugosa*	Rhodophyta	50.50	8.44	TFC-Phyc 16447
*Lobophora dagamae*	Ochrophyta	45.58	5.58	TFC-Phyc 16440
*Colpomenia sinuosa*	Ochrophyta	25.30	10.70	TFC-Phyc 16439
*Ulva clathrata*	Chlorophyta	28.70	35.70	TFC-Phyc 16443
*Halopteris scoparia*	Ochrophyta	60.75	5.91	TFC-Phyc 16442

Each species was gently washed with deionized water, dried at room temperature, and ground into a powder. Seaweed extracts were prepared by adding the dried macroalgal biomass to 150 mL of distilled water (600 mL flask) and sonicating (J.P. Selecta Ultrasounds 3000513, Abrera, Spain) for 30 min at room temperature, after which the mixture was centrifuged to separate the extract from the biomass. This process was repeated three times, then the three extractions were pooled and after removal of solvent by a rotary evaporator (Büchi R-200, Flawil, Switzerland), the extracts of each species were obtained. **Table 1** shows the species, the quantity of dry algae and the yield of each extract.

### 2.2 Extract characterization by NMR (Nuclear Magnetic Resonance)

Each extract was analyzed by NMR using D_2_O as solvent. We measured ^1^H NMR and ^13^C NMR, HSQC (Heteronuclear Single Quantum Coherence), HMBC (Heteronuclear Multiple Bond Correlation) and COSY (Correlated Spectroscopy) spectra using a Bruker Avance II-500 instrument (Bruker, Karlsruhe, Germany) operating at 500 MHz for ^1^H NMR and at 125 MHz for ^13^C NMR. The use of standard Bruker software (TOpSpin 2.1, Bruker, Karlsruhe, Germany) aided the provision of two-dimensional NMR spectra.

### 2.3 Plant material and experimental conditions

Tomato (*Solanum lycopersicum*) var. Robin seedlings were obtained from a local nursery. We sowed tomato seeds in standard 150-cell tomato seedling trays using universal substrate and an automatic sowing machine to ensure germination and growth uniformity. When the seedlings reached the two true leaf stage (two weeks), we started the experiment. The seedling trays were then transferred to a growth chamber with controlled conditions: temperature 24 ± 2°C, photoperiod 16–8 h, humidity 65 ± 2%, and irradiance 300 µmols/m^2^s. All plants received a half-strength Hoagland solution as water supply (Hoagland and Arnon, 1950). Kelpak™ (BASF, Germany) and Sealgae™ (Biovert, Spain) were purchased from a local supplier.

### 2.4 Treatments and water-deficit assays

**Figure 1:**
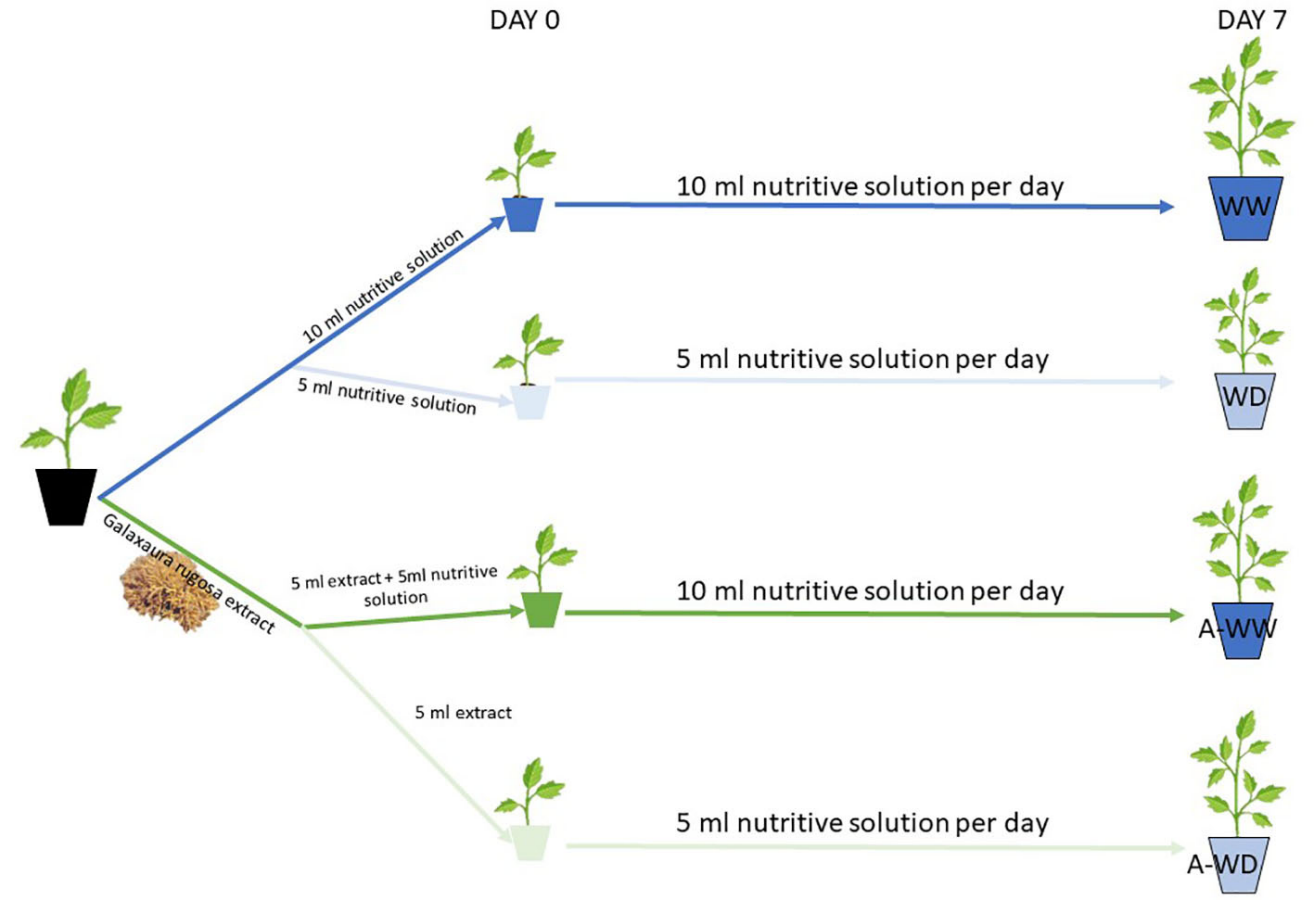
Treatments and experimental set-up. WW and WD mean well-watered and water deficit treatments, respectively. A-WW and A-WD mean *G. rugosa* treatment in well-watered and *G. rugosa* treatment in water deficit conditions, respectively.

All water deficit growth tests were carried out over 7 days according to the method described by Jiménez-Arias et al. (Jiménez-Arias et al., 2022). Water stress was induced by watering at 50% field capacity with a half-strength Hoagland solution, compared to control plants well-irrigated at 100% field capacity. All treatments consisted of twenty plants (N=20). Results are the mean of three independent experiments (see schema of the experimental set up in **Figure 1**).

Plant treatment was conducted by dissolving the various algal extracts at 1% w/v in 100 ml distilled water and adding 5 ml directly to the root system, except for the control treatment which received 5 ml of a half-strength Hoagland solution.

### 2.5 Biomass measures and stress index calculations

Seedling biomass was calculated at the beginning and end of the water deficit period (7 days). We removed the seedlings from the cell trays, carefully washed their roots under water to remove peat, then oven-dried them at 70°C for three days. Relative growth rate (RGR) was estimated according to the formula: RGR = (lnW2 - lnW1)/(t2 - t1), where W1 and W2 are the dry weights of the seedling at times t1 and t2 (the beginning and end of the water deficit, respectively) (Jiménez-Arias et al., 2015). Various indexes were calculated using the weight of the plants at 7 days, such as the stress susceptibility index (SSI) (Ganança et al., 2015), stress tolerance index (TSI) (Farshadfar et al., 2013), relative growth rate (RGR), and plant water use efficiency (WUEp) (Jiménez-Arias et al., 2022).

### 2.6 Gas exchange measurements

The fully developed leaves (N = 30) were subjected to gas exchange analyses. Photosynthesis (Pn), intracellular CO_2_ (Ci), stomatal conductance (gs) and transpiration rate (E) were measured on the attached leaves using a portable infrared gas analyzer (LCPro, BioScientific Ltd., Hoddesdon, UK). Measurements were at ambient CO_2_ concentration, photosynthetic photon flux density (PPFD) of 1000 µmol m^-2^ s^-1^ (optimized with a light curve) and cuvette airflow of 500 ml min^-1^. The values for instantaneous water use efficiency (iWUE) and intrinsic water use efficiency (intWUE) are the ratios between Pn/E and Pn/gs, respectively (Seibt et al., 2008). The ratio between Pn and Ci was also calculated.

### 2.7 Induction of the luciferase reporter in Arabidopsis transgenic seedlings

We grew pMAPKKK18-LUC *Arabidopsis thaliana* seedlings (Vaidya et al., 2017) in 24-well plates (25-35 seeds per well) filled with 1 ml of MS medium supplemented with 1% agar for 7 days (d). Subsequently, Arabidopsis seedlings were treated with mock, 25 μM ABA or 0.5% (w/v) *G. rugosa* extract for 24 h in a solution containing 100 μM luciferin (potassium salt, GoldBio). Seedlings were incubated for 24 h and luminescence was recorded with a LAS-3000 imager (Fujifilm) equipped with a CCD camera using 2 min exposures. We converted eight-bit images to rainbow false color and quantified using Fiji. The experiment was repeated at least twice.

### 2.8 qRT-PCR analysis of ABA-responsive genes in tomato

Ten-day-old tomato seedlings (cv. Moneymaker) were treated with mock or 0.5% G. rugosa extract for 6. Total RNA was extracted using a NucleoSpin RNA plant kit. Synthesis of cDNA and quantitative real-time PCR (qRT-PCR) analyses were performed as described by González-Guzmán et al. (2014). cDNAs corresponding to the ABA-responsive genes *Sl02g084850 (SlRAB18)* and *Sl06g067980 (SlLEA)* genes were amplified using the same primers they used (González-Guzmán et al., 2014). Expression was normalized using values obtained with *Sl06g009970 (SlEF1a)*.

### 2.9 Quantification of ABA

The *G. rugosa* extract was dissolved in 80% methanol-1% acetic acid containing internal standards and mixed by shaking for one hour at 4°C. The extract was stored overnight at -20°C, centrifuged and the supernatant dried in a vacuum evaporator. The dried residue was dissolved in 1% acetic acid and passed through an Oasis HLB (reverse phase) column as described in (Seo et al., 2011). To quantify the hormone ABA, the dried eluate was dissolved in 5% acetonitrile-1% acetic acid and the hormone separated using an autosampler and reverse phase UHPLC chromatography (2.6 µm Accucore RP-MS column, 100 mm length x 2.1 mm i.d.; ThermoFisher Scientific) with a 5 to 50% acetonitrile gradient containing 0.05% acetic acid, at 400 µL/min for 21 min. The hormone was analyzed by selected ion monitoring (SIM) with a Q-Exactive mass spectrometer (Orbitrap detector; ThermoFisher Scientific), and its concentration in the extract determined using embedded calibration curves and the Xcalibur 4.0 and TraceFinder 4.1 SP1 programs. The internal standard for quantification was the deuterium-labeled hormone.

### 2.10 Statistical analyses

After the data normality and homoscedasticity were checked a one-way ANOVA TEST (Duncan´s *post hoc*, IBM-SPSS24 statistical software package) was used to analyze the differences at p values<0.05 between treatments in all the measures studied.

## 3 Results

### 3.1 Seaweeds

The specimens included two species from the phylum Rhodophyta (red algae), namely *Bonnemaisonia hamifera* Hariot and *Galaxaura rugosa* (J. Ellis & Solander); two species from the phylum Chlorophyta (green algae), namely *Dasycladus vermicularis* (Scopoli) Krasser and *Ulva clathrata* (Roth) C.Agardh; and five species from the phylum Ochrophyta (brown algae), namely *Cystoseira foeniculacea* (Linnaeus) Greville, *Cystoseira humilis* Schousboe ex Kützing, *Lobophora dagamae* C.W.Vieira, *Colpomenia sinuosa* (Mertens ex Roth) Derbès & Solier, and *Halopteris scoparia* (Linnaeus) Sauvageau (Guiry and Guiry, 2023). Therefore, our study included representative species from all three main macroalgal groups.

### 3.2 Chemical analysis of crude extracts

The comprehensive metabolic profile analysis of each crude extract was carried out by ^1^HNMR and ^13^C NMR, COSY, HSQC and HMBC experiments (**Supplementary Material**). The main metabolites detected in each extract are listed in **Table 2**, together with their ^1^H and C-13 chemical shifts. The presence of each metabolite was confirmed by comparing its spectroscopic data with those previously described in the literature. NMR spectra analysis of the extract of *B. hamifera* in D_2_O indicated that this extract contains a complex mixture of metabolites, among which floridoside (Ye et al., 2013) appeared as the major metabolite present. The HSQC correlations of signals at δ_C_ 103.0; 98.5; 96.4; 95.9 and 92.1 ppm, with protons at δ_H_ 4.44 (d, 7.6); 4.90 (brs); 4.60 (d, 7.4); 4.66 (d, 7.6) and 5.25 (d, 3.8) ppm respectively, indicate the presence of other saccharides in minor amounts. Other metabolites detected were: isethionic acid (Barrow et al., 1993), acetic acid (Sciubba et al., 2020), formic and lactic acid (Ponder and Richards, 1993) and the amino acids alanine (Sciubba et al., 2020), valine (Ryu et al., 2016), threonine (Salvador et al., 2022) and N,N-dimethyltaurine (Blunden et al., 1986).

**Table 2:** Low-Molecular-weight metabolites detected in the algal extracts and NMR Data in D_2_O [^1^H 500 MHz, ppm, ^13^C 125 MHz D_2_O].

Metabolite	δ_H_ (*J* in Hz)	δ_C_	Species
Alanine	3.82 (m), 1.50 (d, 7.4)	50.7, 16.1, 175.7	*B. hamifera*
Valine	3.63 (m), 2.33 (m), 1.00 (d, 7.8), 1.06 (d, 7.8)	66.2, 28.9, 16.6, 17.9, 181.5	*B. hamifera*
Threonine	3.60 (m), 4.27 (m), 1.33(d)	60.5, 66.1, 20.3	*B. hamifera*
Glycine betaine	3.92 (s), 3.23(s)	67.4, 53.7 169.3	*B. hamifera*
Glycine betaine aldehyde	4.01 (m), 3.23 (s)	77.0, 53.2, 175.7	*G. rugosa* *U. clathrata* *H. scoparia*
Other betaine	3.29 (s)	66.5, 53.3	*B. hamifera* *L. damagae* *U. clathrata* *H. scoparia*
N,N,N-trimethyl taurine	3.50 (m), 3.75 (m), 3.23 (s)	44.8, 61.5, 53.2	*G. rugosa*
N,N-dimethyltaurine	3.38 (m), 3.43 (m), 2.86 (s)	45.4, 53.4, 43.3	*B. hamifera* *G. rugosa*
N-monomethyltaurine	3.30 (m), 3.48 (m), 2.78 (s)	48.9, 44.3, 33.4	*G. rugosa*
Taurine	3.30(t), 3.45(t)	46.9, 35.7	*G. rugosa*
Isethionic acid	3.18 (t), 3.97 (t)	52.8, 57.0	*B. hamifera* *G. rugosa* *H. scoparia* *C. humilis* *D. vermicularis*
Acetic acid	1.94 (s)	23.1, 181.4	*B. hamifera*
Formic acid	8.47(s)		*B. hamifera* *G. rugosa* *C. sinuosa*
Lactic acid	4.15 (m), 1.32 (d, 6.8)	74.2, 22.9, 182.2	*B. hamifera* *C. foeniculacea*
Citric acid	2.54(d), 2.67(d)	48.5, 78.1, 182.1, 184.7	*C. foeniculacea* *C. humilis* *L. dagamae*
3-dimethylsulfoniopropionicate	2.74 (dd, 7.1, 7.1), 3.46 (dd, 7.1, 7.1), 2.92 (s)	39.2, 29.1, 177.0, 25.8	*U. clathrata*
3-hydroxybutyrate	2.36 (dd, 6.4, 14.6); 2.46 (dd, 7.7, 14.6), 4.21 (m), 1.26 (d, 6.4)	46.6, 65.8, 21.9, 180.6	*C. sinuosa*
2,3-dihydroxypropanesulfonate	3.05 (dd, 8.0, 14.5), 3.12 (dd, 3.5, 14.5), 4.18 m, 3.60 (dd, 6.5, 11.3), 3.70 (dd, 4.6, 11.3)	53.7, 68.1, 64.7	*H. scoparia* *U. clathrata*
Floridoside	5.17(d, 3.7), 3.85(m), 3.93 (m), 4.02 (m), 4.13 (m), 3.77 (m), 3.85(m)	98.1, 68.5, 69.3, 69.2, 71.8, 61.2, 61.4, 78.7, 61.4	*B. hamifera* *G. rugosa*
Other saccharides	4.44 (d, 7.6); 4.90 (brs); 4.60 (d, 7.4); 4.66 (d, 7.6); 5.25 (d, 3.8)	103.0; 98.5; 96.4; 95.9; 92.1	*B. hamifera*
α-D-fructofuranose 1,2’:2,3’β-D- dianhydride	4.06 (d, 13.6), 3.77 m, 4.17 (d, 4.7), 3.90 m, 4.12 (ddd, 2.9, 6.2, 6.2), 3.83 (dd, 3.3, 12.8), 3.66 m, 3.71 m; 3.66 m, 4.34 (d, 7.1), 4.68 (dd, 7.5; 7.5), 3.72 m, 3.90m; 3.77 m	60.0, 104.6, 82.2, 76.4, 82.9, 62.1, 64.4, 102.4, 80.0, 73.3, 81.2, 61.6	*D. vermiculata*
Mannitol	3.85 (dd, 1.9, 11.8), 3.66 (dd, 6.1, 11.8), 3.75 (m); 3.79 (m)	63.2, 70.8, 69.2	*C. foeniculacea* *C. humilis* *L. dagamae*
Phloroglucinol monosulfate	6.34 (s), 6.25 (s)	152.7, 100.7, 158.2, 100.9	*H. scoparia*

The 1D and 2D NMR spectra analysis of *G. rugosa* extract in D_2_O showed that this extract also contained a complex mixture of metabolites, including N,N,N-trimethyl taurine (King et al., 1982), N,N-dimethyltaurine, N-monomethyltaurine (McCusker and Klinman, 2009) and taurine (Ye et al., 2013), as well as isethionic acid and floridoside. The NMR data of N-monomethyltaurine and taurine are consistent with those of standard commercial products. Formic acid was also detected.

NMR data for *D. vermicularis* extract show the presence of α-D-fructofuranose 1,2’:2,3’β-D- dianhydride (DFAIII) as the major compound. DFA III has been isolated as a by-product of inulin, obtained from acid hydrolysis, pyrolysis or an enzymatic reaction of inulin (Uchiyama, 1982; Blize et al., 1994), but has also been described as a natural product isolated from *Lycoris radiata* (Li et al., 1997). Although inulin has been found in the green alga *Ulva lactuca* (Aguilera Morales et al., 2018) and inulin-type oligosaccharides have been found in the green alga *Acetabularia crenulata* (Bourne et al., 1972), to the best of our knowledge this is the first time that this metabolite has been found in a marine organism. The ^1^H NMR spectrum of *D. vermicularis* extract also shows low intensity signals at δ_H_ 7.96 (s) and 7.40 (s) ppm in the aromatic region, indicating the presence of sulfated coumarins such as dasycladins A and B and signals at δ_H_ 7.92 (d, 10.1) and 7.36 (d, 10.1) ppm and δ_H_ 7.32 (d, 8.9) and 7.27 (d, 8.9) ppm indicating the presence of 4-(sulfooxy)benzoic acid and 4-(sulfooxy)phenylacetic acid respectively (Hartmann et al., 2018). Another metabolite detected at low levels was isethionic acid.

The 1D and 2D NMR spectra analysis of *U. clathrata* extract in D_2_O indicated that 3-dimethylsulfoniopropionate (Blunden et al., 1992) is the major compound present in this extract that dissolves in D_2_O. Also, 2,3-dihydroxypropanesulfonate (Edmonds and Francesconi, 1983) can be detected in the crude extract of *U. clathrata*.

The extracts of *C. foeniculacea*, *C. humilis* and *L. dagamae* in D_2_O are very similar, mannitol (Yeşil and Akgül, 2022) and citric acid (Pinto et al., 2021) were found, with mannitol being the major constituent of the two in all three extracts. Lactic acid was also detected in the *C. foeniculacea* extract. While the HSQC spectrum of *C. humilis* shows a correlation between the signal at δ_H_ 4.92 (d, 3.9) ppm and the signal at δ_C_ 98.50, indicating the presence of saccharides, and isethionic acid was again detected. The *L. dagamae* extract also shows signals at δ_C_ 102.7: 102.4 and 98.1 ppm, which correlate in the HSQC spectrum with protons at δ_H_ 4.92: 4.80 and 4.53 ppm respectively, indicating the presence of other saccharides in minor amounts.

The 1D and 2D NMR spectra analysis of *C. sinuosa* extract in D_2_O indicated that 3-hydroxybutyrate (Zweifel et al., 2009) is the major compound present in this extract, which dissolves in D_2_O. Another minor metabolite detected by NMR is formic acid.

By contrast, 1D and 2D NMR spectra analysis of *H. scoparia* extract in D_2_O indicated that isethionic acid is the main compound in this extract dissolving in D_2_O. Other metabolites detected by NMR are: 2,3-dihydroxypropanesulfonate and phloroglucinol monosulfate (Glombitza and Knöss, 1992).

Betaines and sulphonium compounds are common in polar extracts of marine algae (Blunden et al., 1986; Blunden et al., 1992). In addition to the substances mentioned above, three betaines were detected in five of the nine algae according to the HMBC correlations of the N-methyl groups. The HMBC correlations of the N-Me protons at δ_H_ 3.26 (s) with both the methyl signal at δ_C_ 53.6 ppm and the methylene signal at δ_C_ 64.6 ppm indicate the presence of glycine betaine (Blunden et al., 1992) in *B. hamifera* extract. The HMBC correlations of the N-Me protons at δ_H_ 3.21 (s) with the methyl carbons at δ_C_ 53.5 ppm and the methylene signal at δ_C_ 78.0 ppm point to another betaine, probably glycine betaine aldehyde (Blunden et al., 1992) in *G. rugosa*, *U. clathrata* and *H. scoparia* extracts. Finally, HMBC correlations of the N-Me protons at δ_H_ 3.29 (s) with methyl carbons at δ_C_ 54.3 ppm and with the methylene signal at δ_C_ 67.0 ppm suggest the presence of another unidentified betaine in *B. hamifera*, *U. clathrata*, *L. dagamae* and *H. scoparia* extracts.

### 3.3 Several seaweed extracts improve tomato tolerance to water deficit

**Figure 2:**
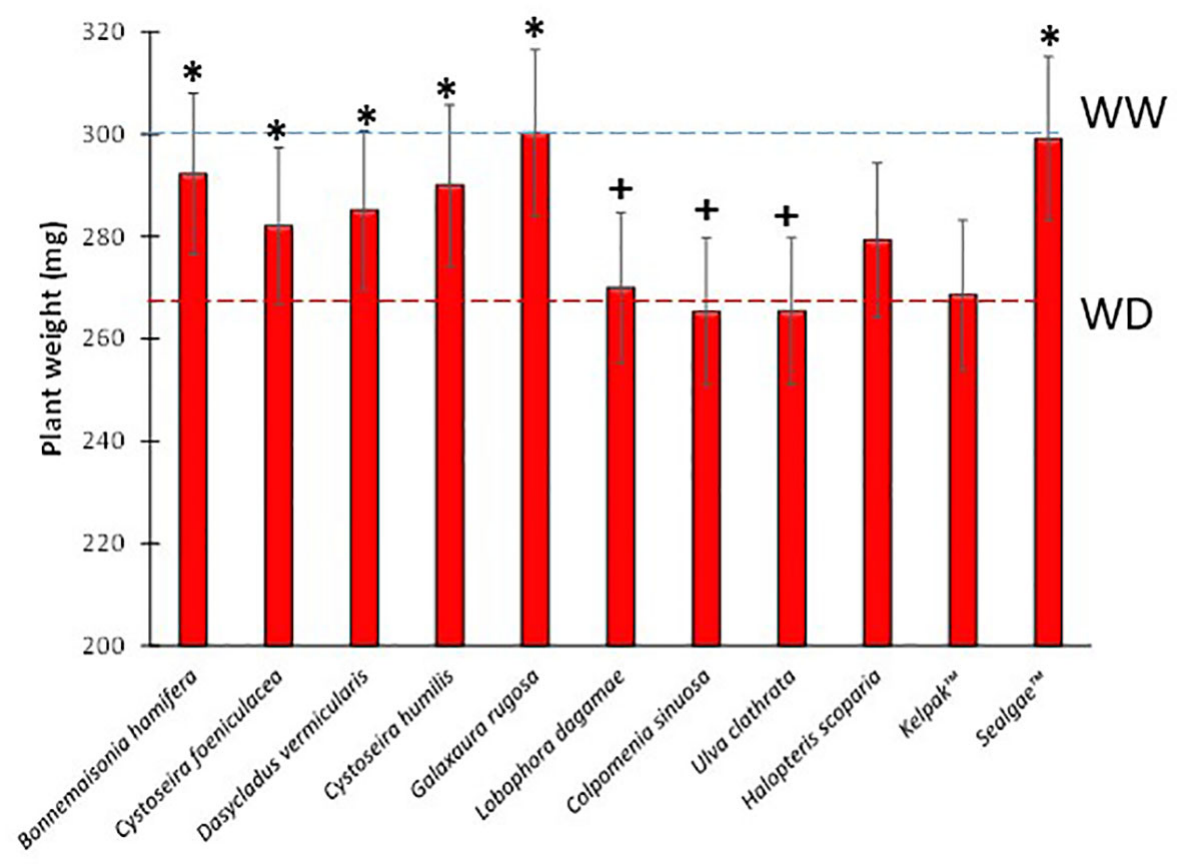
Dry weights of tomato plants treated with different algal extracts under water-deficit stress conditions. Blue and red dashed lines represent WW and WD dry weight average respectively. ^+^ and * means significant differences at p< 0.05 with respect to WW and WD, respectively.

In order to test the biostimulant activity of the nine algal extracts, tomato seedlings were subjected to water deficit conditions (WD, 50% field capacity) after treating their root systems with the different extracts (see **Table 3**). Seedling dry weight of WD treatment was reduced by 11.2% after 7 days (**Figure 2**).

**Table 3:** Relative growth rate and water use efficiency index studied in the different treatments under water deficit conditions.

Treatment	RGR	WUEp
WW	0.16	2.8
WD	0.14	4.7
*Bonnemaisonia hamifera*	0.16	5.6 *
*Cystoseira foeniculacea*	0.15	5.3
*Dasycladus vermicularis*	0.15	5.4 *
*Cystoseira humilis*	0.16	5.5 *
*Galaxaura rugosa*	0.16	5.8 *
*Lobophora dagamae*	0.15	4.9
*Colpomenia sinuosa*	0.14	4.8
*Ulva clathrata*	0.14	4.8
*Halopteris scoparia*	0.15	5.2
*Kelpak*™	0.15	4.9
*Sealgae*™	0.16	5.8*

WW represents well-watered conditions (100% field capacity) and WD water deficit conditions (50% field capacity) as indicated in Materials and Methods.

*Mean significant differences from WD. Kelpak™ (BASF, Germany) and Sealgae™ (Biovert, Spain) were purchased from a local supplier.

Seedlings exposed to WD also showed significant differences in water use efficiency (WUEp), compared to the well-watered (WW) plants, which increased by 66.3% (**Table 3**). However, the seedling dry weight of *B. hamifera*, *D. vermicularis*, *C. humilis*, and *G. rugosa* extract treatments was significantly higher compared to WD, comparable to the commercial extract Sealgae™ used as a positive control (**Figure 2**). Interestingly, treatment with *B. hamifera*, *D. vermicularis*, *C. humilis*, and *G. rugosa* extracts caused a significant increase in WUE compared to mock-treated WD plants (**Table 3**). Furthermore, drought stress indexes, RGR and WUE were also significantly higher for the above treatments (**Table 3**).

### 3.4 *Galaxaura rugosa* extract improves tomato plant tolerance of water deficit

**Figure 3:**
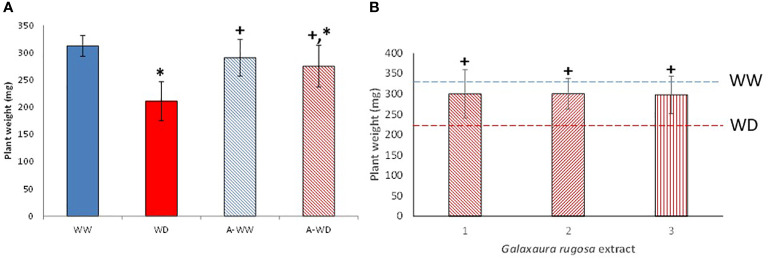
Dry weights of tomato plants treated with *G. rugosa* extract under well-watered and water-deficit stress conditions. **(A)** Dry weights of tomato plants treated with different *G. rugosa* extracts under water-deficit conditions. **(B)** Blue and red dashed lines represent WW and WD dry weight average respectively. ^+^ and * means significant differences at p< 0.05 with respect to WW and WD respectively. 1-3 means *G. rugosa* extracts from different seasons.

After the bioprospection results, *G. rugosa* extract performs best under WD conditions. For this reason, deeper analyses were carried out under WW or WD conditions (**Figure 3A**). Seedlings treated with the extract show a small but non-significant reduction in plant weight under well-watered conditions, while the protective effect is clearly visible under deficit conditions (**Figure 3A**). It is noteworthy that extracts collected in different seasons can replicate this protective behavior (**Figure 3B**). Growth and tolerance index clearly show the tolerance enhancement by root treatment with *G. rugosa* (**Table 4**). Under a low watering regime, treated plants increased in RGR, WUEp, and STI by 31, 53 and 40%, respectively, compared to untreated seedlings, showing an 83% decrease in sensitivity as SSI indicated.

**Table 4:** Relative growth rate and stress indexes attained with the *Galaxaura rugosa* treatment under water deficit conditions.

Treatment	RGR	WUEp	SSI	STI
WW	0.18	3.1		
WD	0.12	3.4	1.7	0.7
A-WW	0.17	2.9		
A-WD	0.16	5.2	0.3	1.0

WW and WD mean well-watered and water deficit treatments, respectively. A-WW and A-WD mean *G. rugosa* treatment in well-watered and *G. rugosa* treatment in water deficit treatment, respectively.

**Figure 4:**
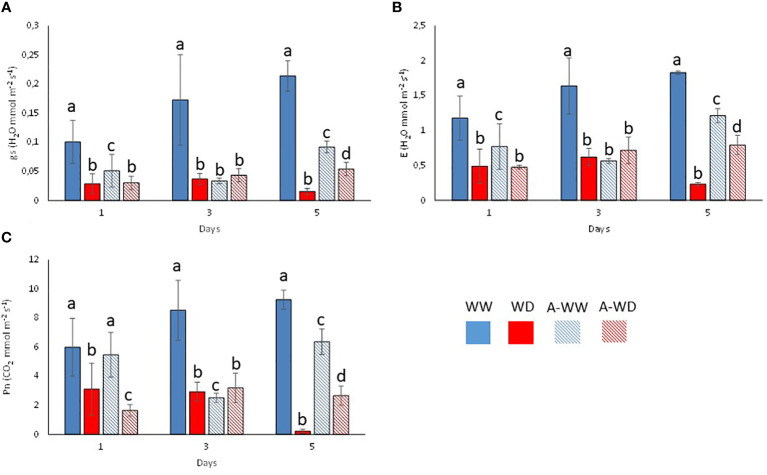
Plant gas exchange measurements and CO_2_ assimilation during the experiment. **(A)** Transpiration (E). **(B)** Stomatal conductance (gs). **(C)** Net photosynthesis (Pn). Bars labeled with letters indicate significant differences at p<0.05. Bars labeled by the same letter did not show significant differences at p<0.05.

**Figure 5:**
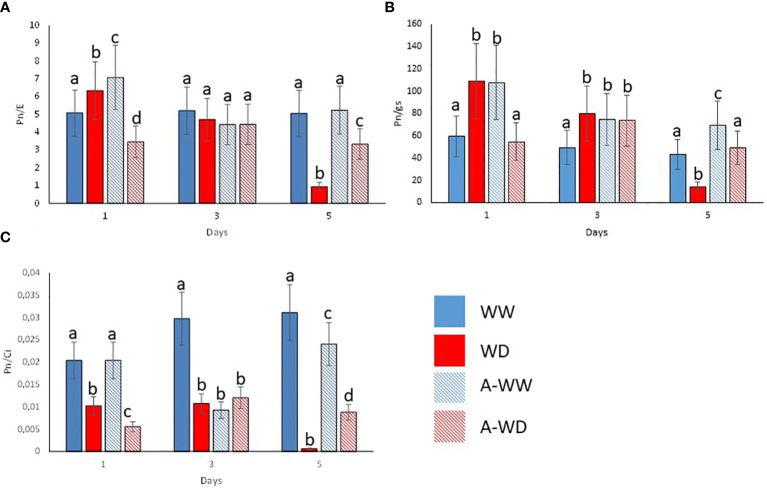
Enhanced WUE and CO_2_ assimilation ratio by *G. rugosa* treatment under WD conditions. **(A)** Instantaneous water use efficiency, Pn/E. **(B)** Intrinsic water use efficiency, Pn/gs. **(C)** Pn/Ci index. Bars labeled with the same letter did not show significant differences at p<0.05. Ratios and standard deviations were obtained from values reported in **Figure 3**.

Mock-treated plants exposed to WD continuously decreased stomatal conductance, transpiration, and net photosynthesis (**Figures 4A–C**). Application of the *G. rugosa* extract led to the recovery of photosynthesis at 5 days after the onset of WD, which also correlated with higher gs and E compared to mock-treated plants subjected to WD (**Figures 4A–C**). Applying the algal extract to well-watered plants also reduced gs, E and Pn, reaching its minimum three days after stress exposure, indicating that the extract’s components likely induce stomatal closure (**Figures 4A–C**). We also calculated the iWUE and int WUE parameters, and the efficiency of CO_2_ assimilation according to the Ci (**Figure 5**). At 3 and 5 days, we observed that plants subjected to WD and treated with the *G. rugosa* extract showed higher instantaneous and intrinsic WUE than mock-treated plants (**Figures 5A, B**). The algal extract also improved the Pn/Ci ratio in A-WD compared to WD plants (**Figure 5C**).

### 3.5 *Galaxaura rugosa* extract induces expression of ABA-responsive genes in *Arabidopsis* and tomato

Seaweed extracts are complex mixtures, so to understand their action mechanisms is challenging. ABA is an important phytohormone for coping with water deficit stress, therefore activation of ABA signaling leads to increased WUE in crops (Mega et al., 2019; Yang et al., 2019; Mao et al., 2022). Recently, an ABA receptor agonist capable of activating ABA signaling in tomato was reported to have biostimulant activity against water deficit stress (Jiménez-Arias et al., 2023). Thus, to further understand the action mechanism of the seaweed extracts reported here, we focused on a sample that showed strong biostimulant potential, i.e., *G. rugosa*, to establish whether it could activate ABA signaling in either *Arabidopsis thaliana* (arabidopsis) or tomato. We first measured the ABA concentration in the *G. rugosa* extract as described in the Materials and Methods section, finding it to be 0.45 ± 0.12 nM. This ABA concentration was used in subsequent mock-treated samples to maintain the background effect due to residual ABA in that extract.

**Figure 6:**
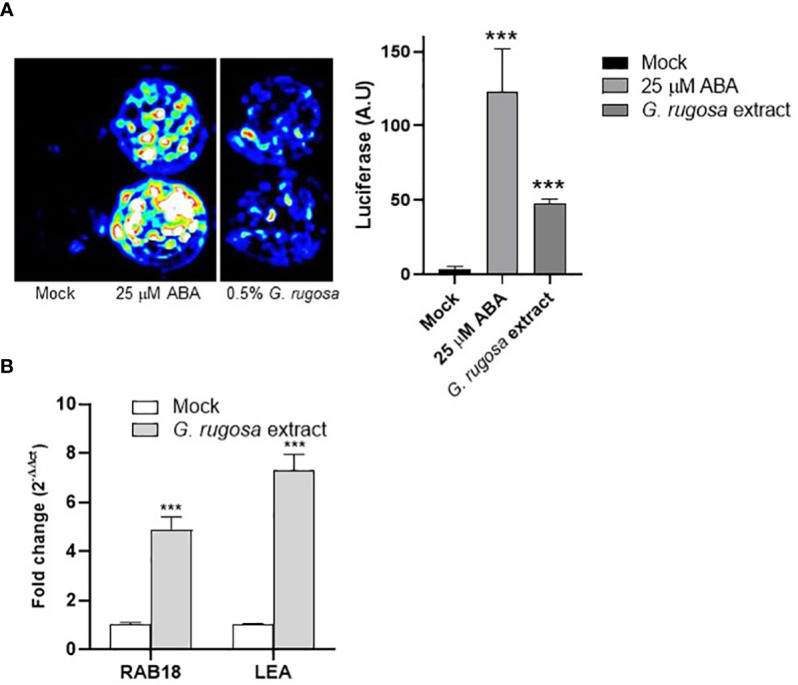
Induction of ABA-responsive genes in *Arabidopsis thaliana* and tomato by the *G. rugosa* extract. **(A)** Incubation with *G. rugosa* extract induces expression of luciferase (LUC) in the pMAPKKK18 LUC reporter line. Seedlings of the *Arabidopsis* reporter line were mock-treated with 25 μM ABA or 0.5% *G. rugosa* extract in 24-well plates and imaged with a charge-coupled device (CCD) camera to detect luminescence 24 h later. Quantification of luminescence is shown in the right panel and expressed as arbitrary units (a.u.). Asterisks indicate statistical significance (p<0.01). **(B)** The *G. rugosa* extract upregulates the expression of ABA-responsive genes in tomato. Ten-day-old tomato seedlings were treated with either mock or 0.5% *G. rugosa* extract for 6h. Histograms indicate the relative induction of the *SlRAB18* namely *Sl02g084850* and *SlLEA namely Sl06g067980* genes by the seaweed treatment with respect to mock treatment (value 1). Expression of *SlEF1a* was used to normalize the expression of ABA-responsive genes.

To test the effect on ABA signaling, we used an arabidopsis transgenic line in which the ABA-responsive *MAPKKK18* promoter was fused to the LUC reporter (Vaidya et al., 2017). We incubated arabidopsis seedlings treated with mock, ABA or seaweed extract for 24 h in a solution containing 100 μM luciferin (**Figure 6A**). As a result, we observed induction of the LUC reporter by treatment with a 0.5% *G. rugosa* extract (**Figure 6A**). To test the effect of the extract in wild-type tomato seedlings, we treated tomato seedlings with a 0.5% *G. rugosa* extract for 6 h and assessed the expression of two ABA-responsive genes, namely *Sl02g084850 (SlRAB18)* and *Sl06g067980 (SlLEA)*. As a result, we found that *G. rugosa* extract induced ABA-responsive genes in both arabidopsis and tomato (**Figure 6B**), which may contribute to the protective effect seen in the water deficit experiment.

## 4 Discussion

In recent years, the need to look for environmentally friendly alternatives to protect our crops has become apparent. In the EU at least, measures have been promoted to achieve this goal, recognizing that in order to feed a growing world population and ensure food security we need to optimize yields. One of the most obvious and widespread consequences of climate change and global warming is drought, whose impact on crop yields threatens our future (Summary for Policymakers, 2014). It is therefore important to look for sustainable alternatives to save water in agriculture. One possible solution is the use of biostimulants to increase plant tolerance to water deficit conditions.

From our bioprospection in WD conditions, plants-treated with aqueous extracts of four seaweeds, *B. hamifera*, *D. vermicularis*, *C. humilis* and *G. rugosa* at 0.1 g/L showed significantly higher dry weights compared to mock-treated or plants-treated with the rest of the extracts (**Figure 2**). Given these results, it seems obvious that not all extracts are able to increase plant tolerance under the water deficit conditions to which they were exposed. Therefore, the chemical composition of these extracts is crucial in determining how well they confer tolerance in the conditions tested (Ertani et al., 2018). In this context, there are seaweed-based or organic biostimulant treatments in the literature that show different levels of effectiveness under water deficit or drought conditions, even different extracts from the same seaweed led to different activity (Goñi et al., 2016). For example, in *Betula papyrifera* (paper birch) seedlings exposed to drought, treatment with an organic biostimulant did not improve tolerance to drought stress (Richardson et al., 2004). However, improved tolerance was observed in grapes treated with *Ascophyllum* extracts and exposed to drought. This latter case was attributable to the beneficial effect of these extracts on plant osmotic status and the effect of the betaines and oligosaccharides in their composition (Norrie et al., 2002).

Chemical study of our extracts showed that some substances are common to several species, such as mannitol and isethionic acid. Mannitol is the main constituent in the extracts from *L. dagamae*, *C. foeniculacea* and *C. humilis*. On correlating their mannitol content with extract activity, we conclude it is not be responsible for the activity of *C. humilis* extract, since the other two are not active. Similarly, isethionic acid is the main compound in the inactive *H. scoparia* extract that is also found in *B. hamifera*, *G. rugosa* and *D. vermicularis* extracts, which did improve drought tolerance activity.

We consider that *C. humilis* is active because of a minor metabolite or the synergistic effect of more than one substance. For additional research progress, it would be necessary to carry out a bioguided fractionation of *D. vermicularis*, to see if DFAIII or one of the sulfated coumarins could be responsible for its activity.

The two red algae studied, *B. hamifera* and *G. rugosa*, share some common products such as N,N-dimethyl taurine and floridoside, which latter is a characteristic component of several red algae species (Ekman et al., 1991). In addition to such compounds, analysis of the betaines detected suggests that glycine betaine could be responsible for the activity of *B. hamifera*, since this substance was not detected in any other extract. However, glycine betaine aldehyde and the unidentified betaine were detected not only in the active extract of *G. rugosa*, but also in non-active extracts of *U. clathrata* and *H. scoparia*, so it does not appear that these substances alone are responsible for the activity of the *G. rugosa* extract. We plan to purify the crude extracts following a bioguided fractionation process to examine individual metabolites and determine which are responsible for the observed biological activity, either individually or in combination.

Under our conditions, the *G. rugosa* extract showed the highest osmoprotectant activity under WD stress (**Figure 2**). Interestingly, we also observed that well-watered plants treated with the *G. rugosa* extract showed reduced gs and E compared to mock-treated plants (**Figure 2**). This suggests that the extract induces stomatal closure, which might indicate priming of ABA signaling after the application of the seaweed extract. Although closure of stomata limits the CO_2_ uptake necessary for photosynthesis, one of the first stress responses of plants under drought conditions is indeed to close the stomata to reduce water loss and maintain cell turgor (Mega et al., 2019; Yang et al., 2019; Mao et al., 2022). Recently, the ABA receptor agonist AMF4 was reported to improve CO_2_ assimilation and WUE in plants subjected to WD stress (Jiménez-Arias et al., 2023). Interestingly, the seaweed extract behaved analogously to AMF4 in plants subjected to WD stress (**Figure 3**, **4**). Thus, comparing the gas-exchange values of *G. rugosa*-treated and mock-treated tomato seedlings under WW or WD stress conditions reveals a clear effect of the seaweed extract on the gs and E values, and photosynthesis protection after 5 days of WD. Whereas the gs and E of mock-treated plants were significantly reduced under WD conditions, leading to a dramatic drop in photosynthesis, the *G. rugosa* treatment significantly improved CO_2_ assimilation, relative growth rate and all tolerance and susceptibility indexes compared to WD control plants (**Figures 4**, **5**; **Table 4**). These results show that the seaweed extract appears to partially mimic ABA or ABA receptor agonists’ effect to modulate gs to avoid water loss (**Figure 4A**).

Our results with the *G. rugosa* extract are consistent with previous work using the seaweed *Ascophyllum nodosum* in arabidopsis under drought stress conditions (Santaniello et al., 2017; Rasul et al., 2021). Santaniello et al. (2017) found that plants treated for 5 days with *A. nodosum* seaweed extract showed a partial stomatal closure and reduced gs. The authors suggested that this effect together with the preactivation of ABA-responsive genes and antioxidant system pathways were responsible for plant tolerance to drought stress (Santaniello et al., 2017). Likewise, priming with the biostimulant super fifty (also produced from an *A. nodosum* seaweed *extract*) improved drought tolerance in arabidopsis through the maintenance of higher relative water content and expression of ABA-dependent genes (Rasul et al., 2021). Therefore, we also investigated the possible induction of ABA-responsive genes in an arabidopsis LUC reporter line or tomato seedlings. The residual ABA concentration of the extract (below 0.5 nM) was used as mock-treatment as explained. Still, it was not enough to significantly activate the *pMAPKKK18-LUC* reporter, in contrast to the seaweed treatment (**Figure 6A**). Two tomato ABA-responsive genes were also induced in response to treatment with the *G. rugosa* extract. Thus, activating the ABA pathway probably contributes to the protective effect of the *G. rugosa* extract in plants subjected to water deficit conditions (**Figure 6B**). These results do not exclude protection effects contributed by other mechanisms, for example by compatible solutes (betaines, amino acids, etc.) that favor osmoadjustment processes. However, future studies should address the mechanism whereby certain seaweed extracts induce activation of the ABA pathway (Santaniello et al., 2017; Rasul et al., 2021; this work). Priming of the ABA response by small molecules such as ß-aminobutyric acid involves ABA accumulation, resulting in stomatal closure and activation of the antioxidant defense enzymes (Du et al., 2012). We speculate that some components of the extract might affect ABA biosynthesis or signaling, acting as priming agents. It is also possible that the sensitivity of the stomata to low changes in ABA concentration is increased after seaweed treatment (Santaniello et al., 2017).

The results of this study demonstrate the potential of some of the algae studied as a source of extracts and/or products that biostimulate drought tolerance in tomato plants. This advance prompts us to continue with a more detailed study of the active algae, to determine which substances exert this biostimulatory effect. Synergistic effects aside, the individual activity of the substances identified should be much greater than that of the crude extract and they are likely to have industrial applications.

## Data availability statement

The data presented in the study are deposited in the zenodo.org repository, accession number https://zenodo.org/record/8326436.

## Author contributions

AB and MC contributed to the conception and design of the study. AB, MC and PR acquired the funding; DJ-A, SM-S, JL, NR-R, AC, PR, AB and MC performed the research. DJ-A and AB performed statistical analysis. AB and MC wrote the original draft of the manuscript. DJ-A, AC and NR-R prepared the figures. DJ-A, AC, PR and AB prepared the graphs. All authors contributed to the article and approved the submitted version.
