# Supplementary material for: Biostimulant activity of Galaxaura rugosa seaweed extracts against water deficit stress in tomato seedlings involves activation of ABA signaling
Source: Front Plant Sci. 2023 Sep 14;14:1251442. doi: 10.3389/fpls.2023.1251442 (PMC10538540; doi:10.3389/fpls.2023.1251442)
Supplement: Supplementary file 1 [file DataSheet_1.pdf]

Supplementary Material

Biostimulant activity of *Galaxaura rugosa* seaweed extracts against water deficit stress in tomato seedlings involves activation of ABA signaling

Sarai Morales-Sierra[1], Juan Cristo Luis[1], David Jiménez-Arias[2], Nereida M. Rancel-Rodríguez[3], Alberto Coego[4], Pedro L. Rodriguez[4], Mercedes Cueto[5,*], Andrés A. Borges[5,*]

[1]Grupo de Biología Vegetal Aplicada (GBVA), Departamento de Botánica, Ecología y Fisiología Vegetal, Facultad de Farmacia Universidad de La Laguna. Avenida Astrofísico F. Sánchez s/n, 38206 La Laguna, Tenerife, Spain

[2]Instituto Canario de Investigaciones Agrarias (ICIA), Departamento de Producción vegetal en zonas tropicales y subtropicales. TF-156, 168, 38297 La Laguna, Santa Cruz de Tenerife, Spain

[3]Grupo BotMar-ULL. Departamento de Botánica, Ecología y Fisiología Vegetal, Facultad de Farmacia Universidad de La Laguna. Avenida Astrofísico F. Sánchez s/n, 38206 La Laguna, Tenerife, Spain

[4]Instituto de Biología Molecular y Celular de Plantas, Consejo Superior de Investigaciones Científicas, Universidad Politécnica de Valencia, ES-46022 Valencia, Spain

[5]Departamento de Ciencias de la Vida y de la Tierra, Departamento de Productos Naturales y Sintéticos Bioactivos, Instituto de Productos Naturales y Agrobiología (IPNA-CSIC). Avenida Astrofísico F. Sánchez, 3, 38206 La Laguna, Tenerife, Spain

*** Correspondence:**
A. A. Borges
aborges@ipna.csic.es

M. Cueto
mcueto@ipna.csic.es

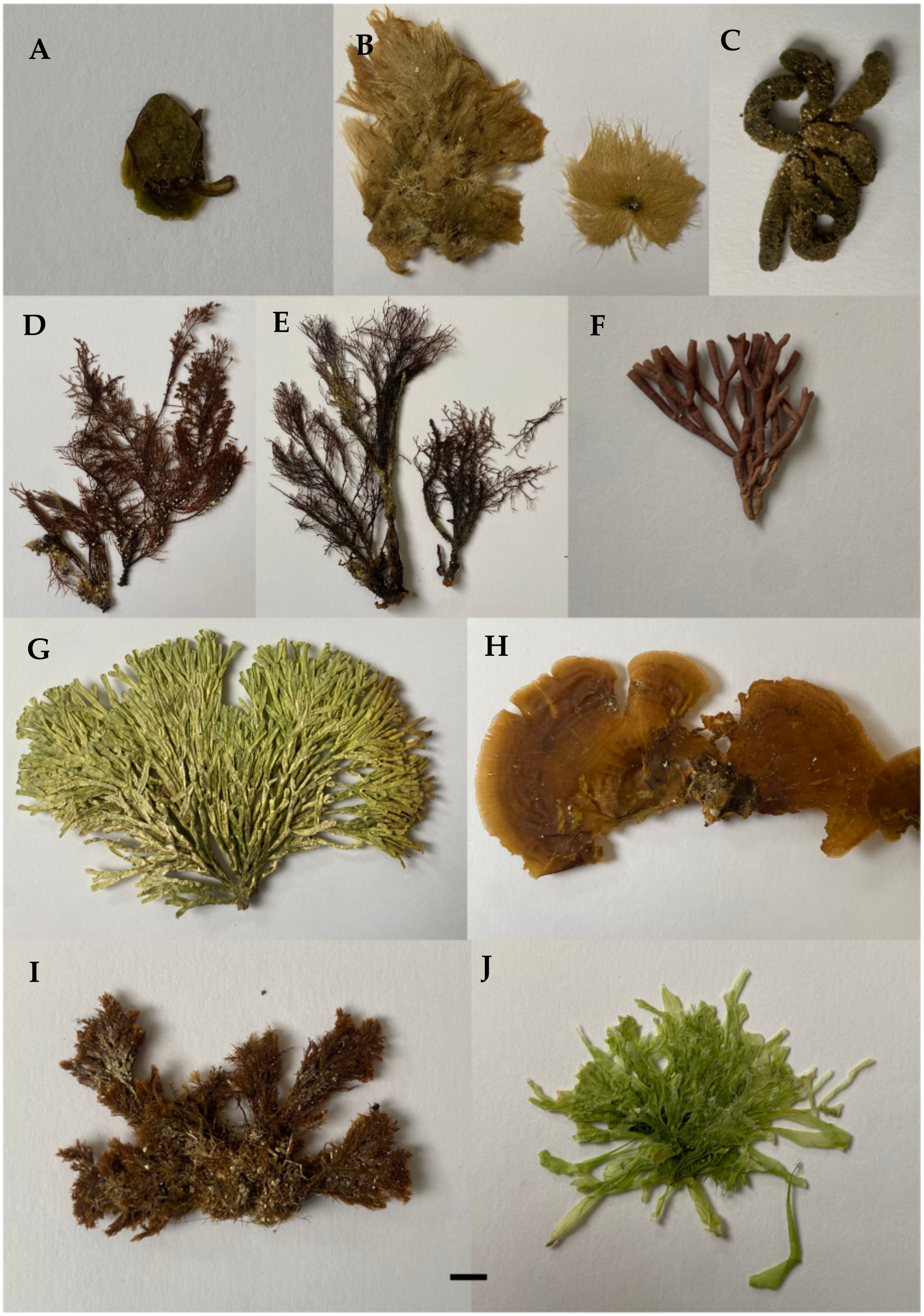

Supplementary Figure 1: Photographic plate illustrating the herbarium sheets of the algae collected during this study, which have been safely preserved and catalogued in the Institutional Herbarium TFC of the University of La Laguna, with their respective herbarium ID numbers. A. TFC-Phyc 16.439 *Colpomenia sinuosa* B. TFC-Phyc 16441 *Bonnemaisonia hamifera* C. TFC-Phyc 16444 *Dasycladus vermicularis*. D. TFC-Phyc 16445 *Cystoseira humilis* E. TFC-Phyc 16446 *Cystoseira foeniculacea* F. TFC-Phyc 16447 *Galaxaura rugosa* (preserved without formaldehyde) G. TFC-Phyc 16447 *Galaxaura rugosa* (preserved with formaldehyde) H. TFC-Phyc 16440 *Lobophora dagamae* I. TFC-Phyc 16442 *Halopteris scoparia* J. TFC-Phyc 16443 *Ulva clathrate*.

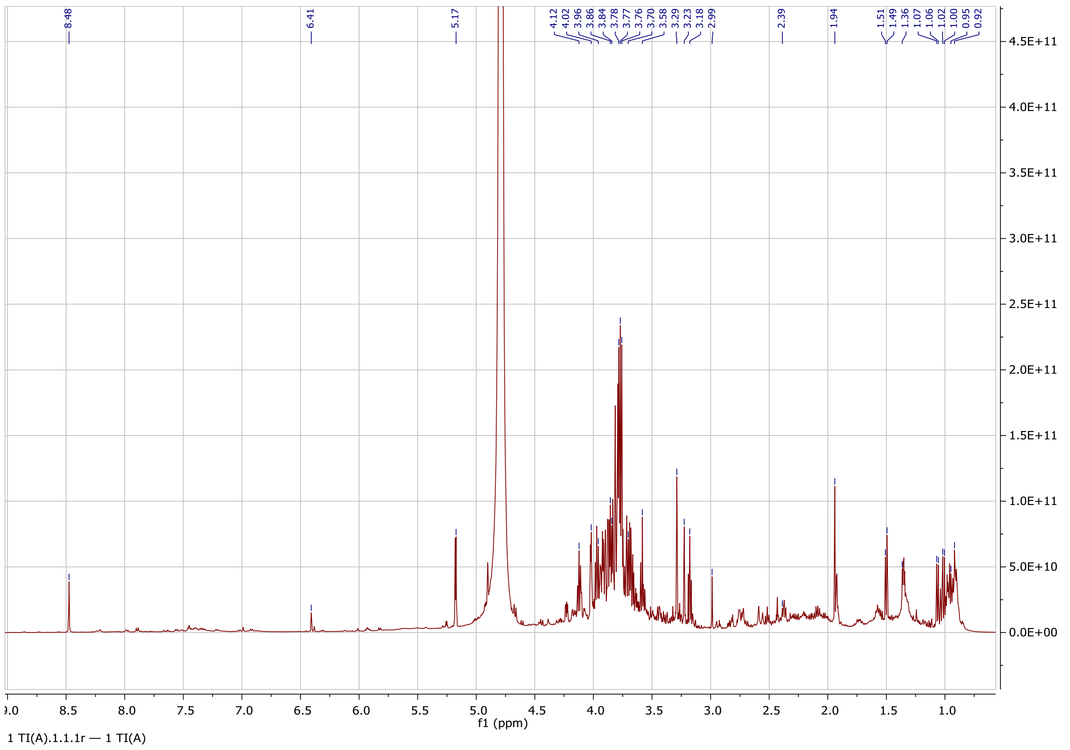

Supplementary Figure 2: ^{1}H NMR spectrum of *Bonnemaisonia hamifera* in D_2O

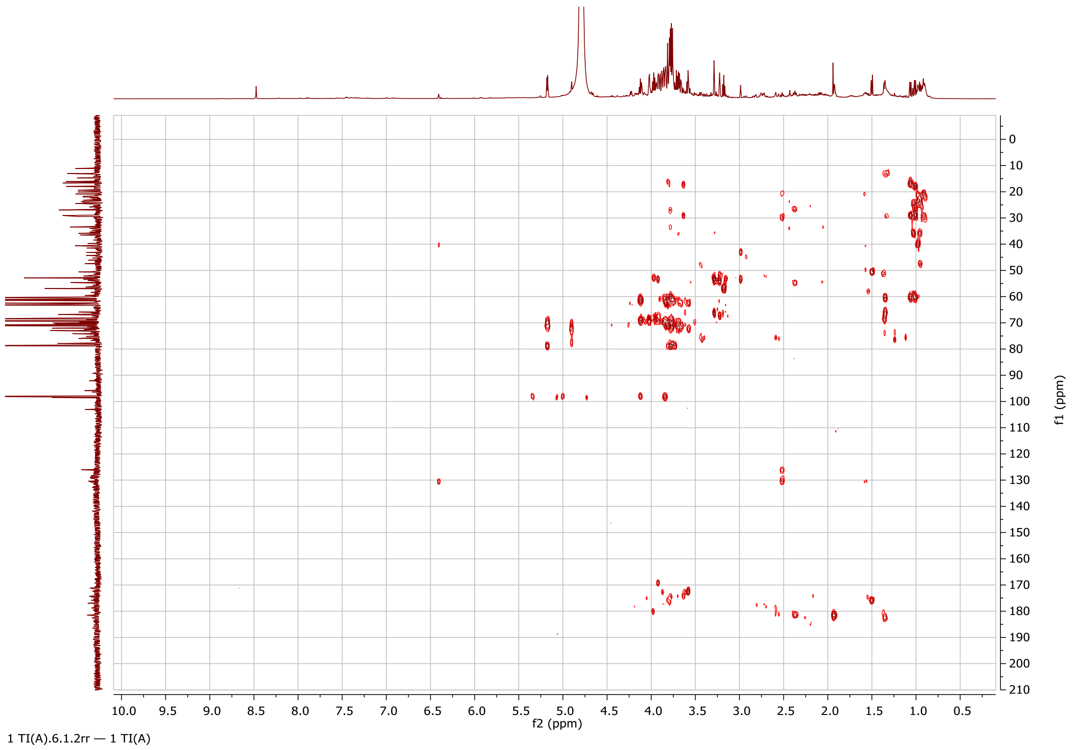

Supplementary Figure 3: HMBC spectrum of *Bonnemaisonia hamifera* in D_2O

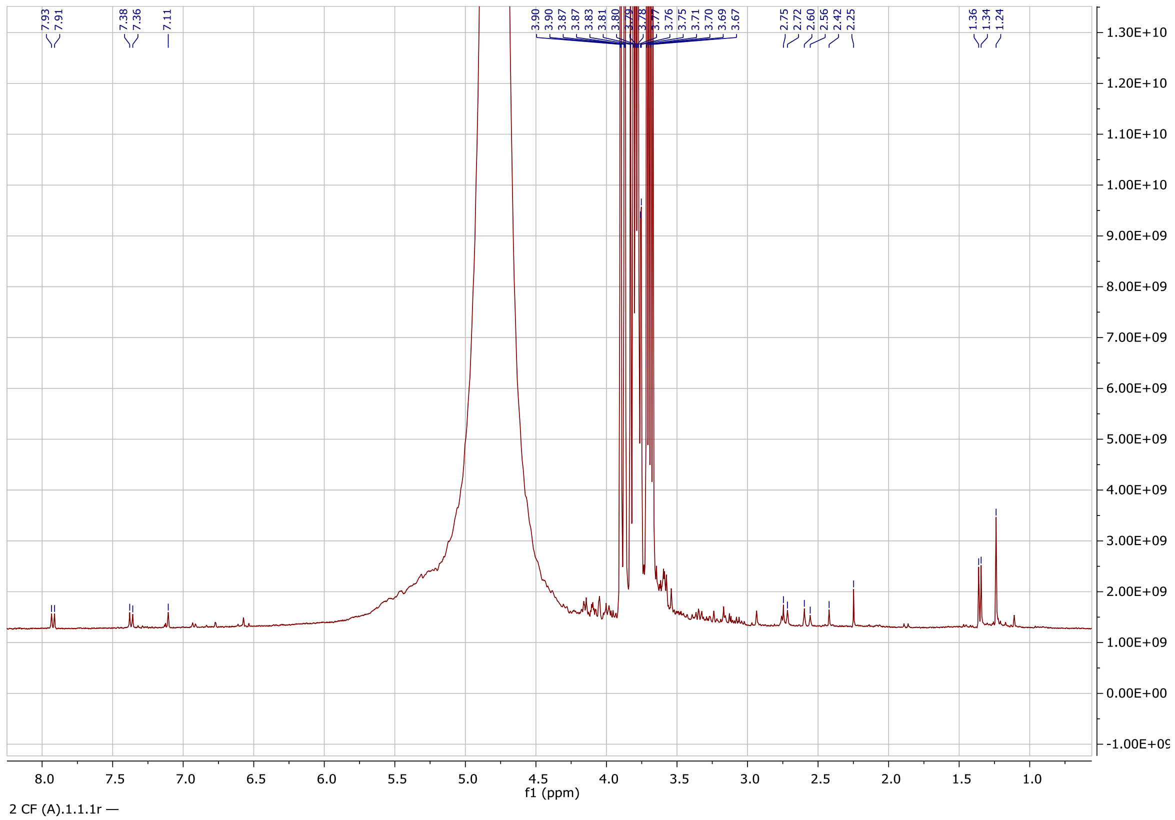

Supplementary Figure 4:^{1}H NMR spectrum of *Cystoseira foeniculacea* in D_2O

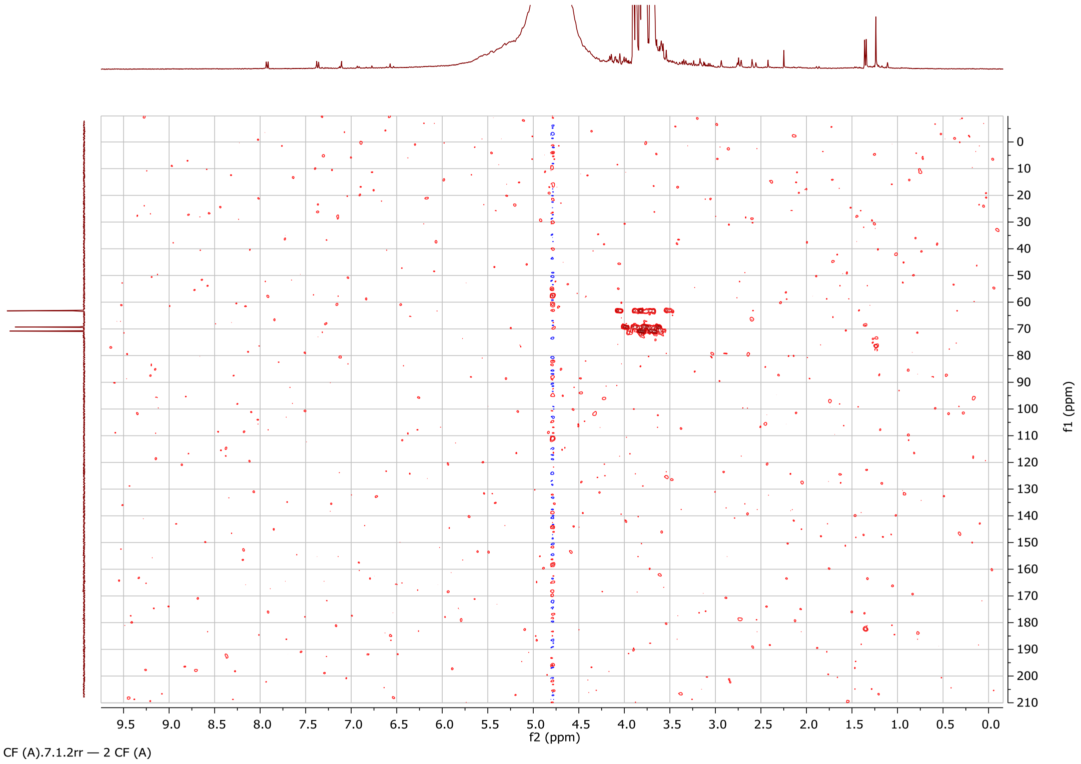

Supplementary Figure 5: HMBC spectrum of *Cystoseira foeniculacea* in D_2O

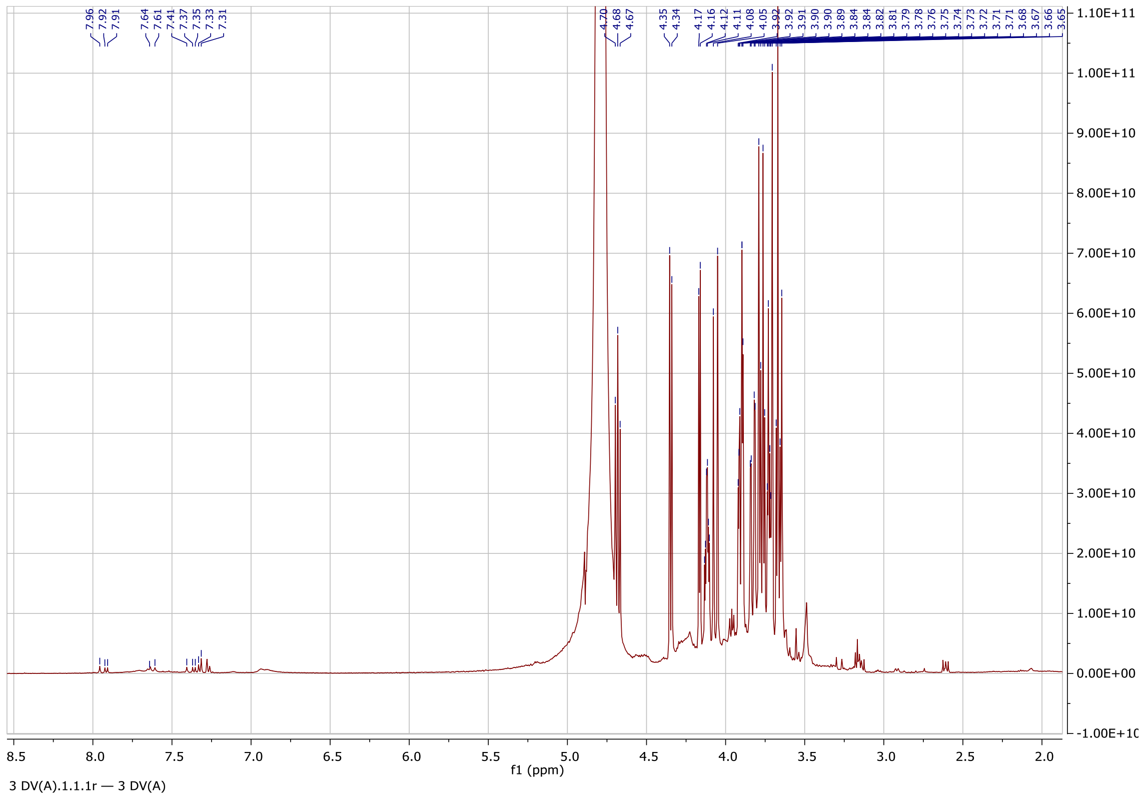

Supplementary Figure 6: ^{1}H NMR spectrum of *Dasycladus vermicularis* in D_2O

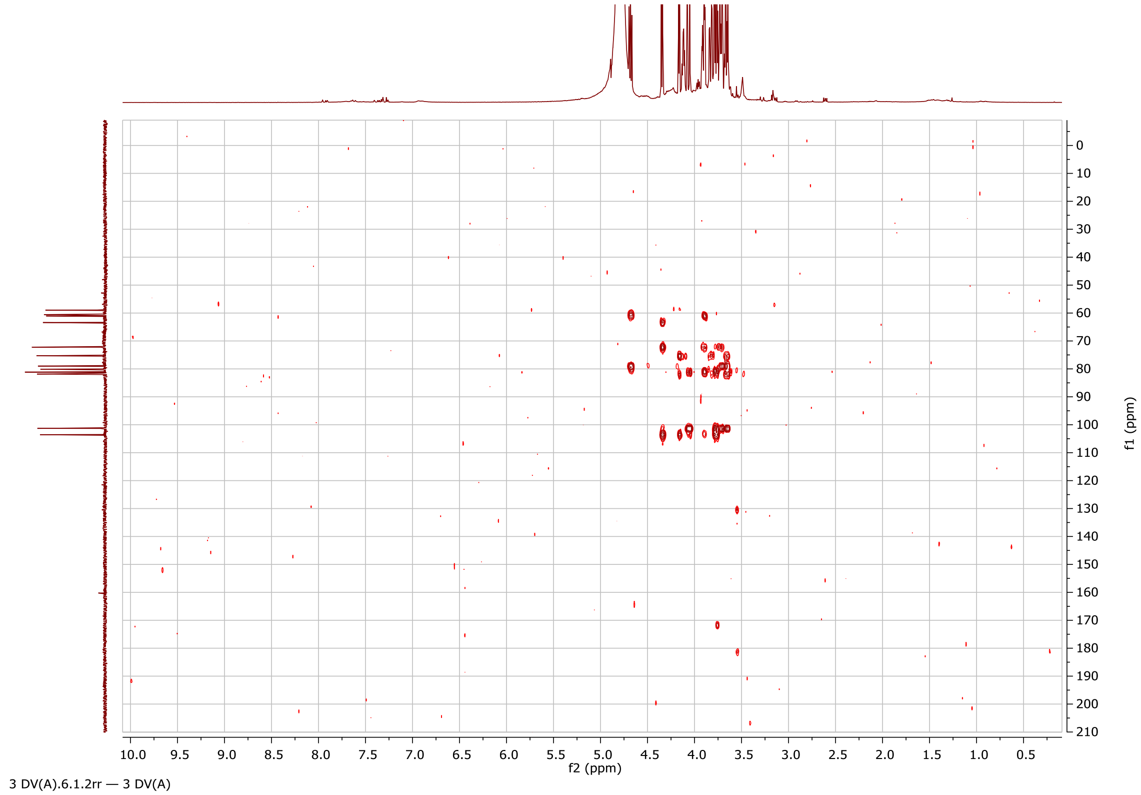

Supplementary Figure 7: HMBC spectrum of *Dasycladus vermicularis* in D_2O

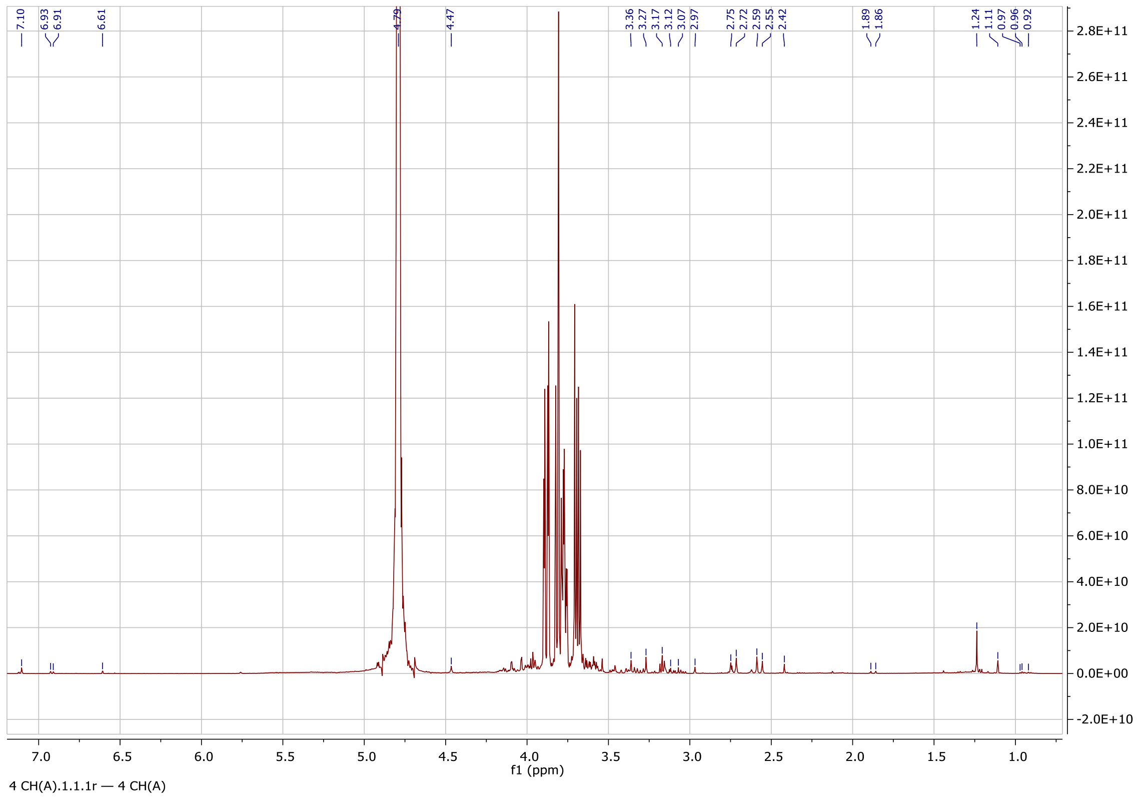

Supplementary Figure 8: ^{1}H NMR spectrum of *Cystoseira humilis* in D_2O

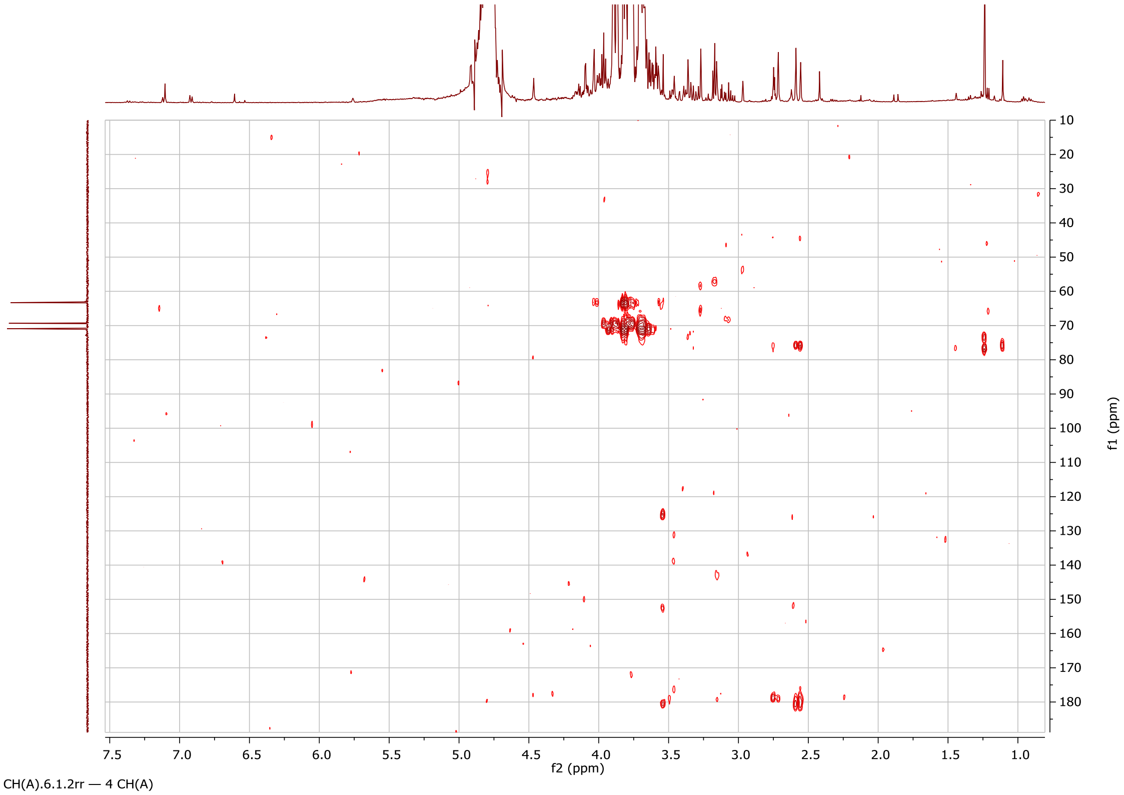

Supplementary Figure 9: HMBC spectrum of *Cystoseira humilis* in D_2O

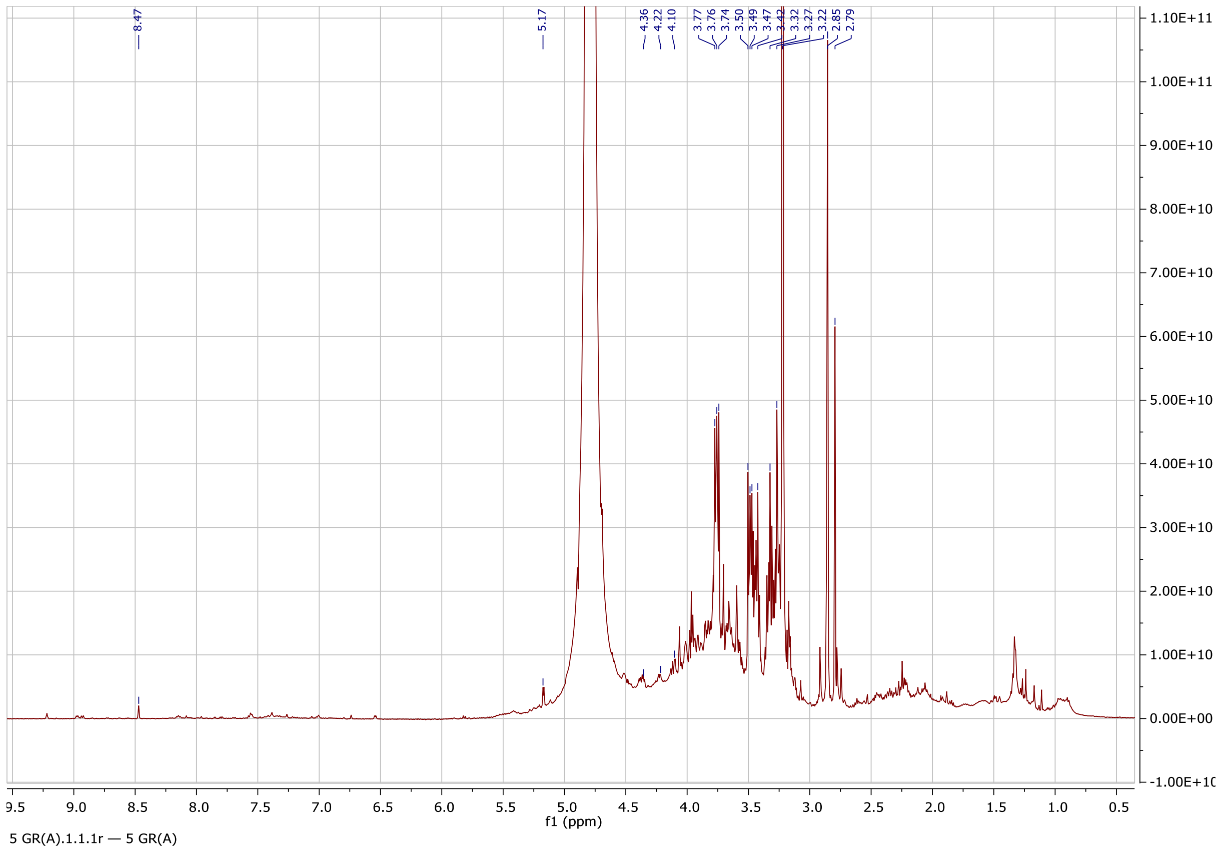

Supplementary Figure 10: ^{1}H NMR spectrum of *Galaxaura rugosa* in D_2O

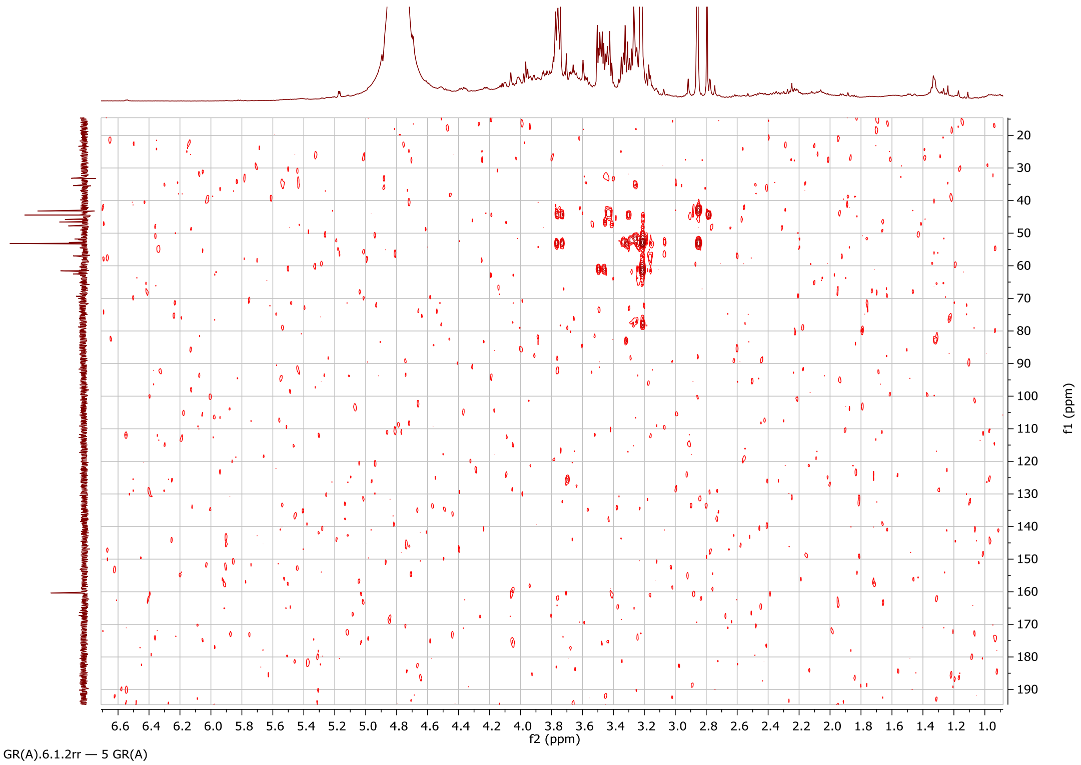

Supplementary Figure 11: HMBC spectrum of *Galaxaura rugosa* in D_2O

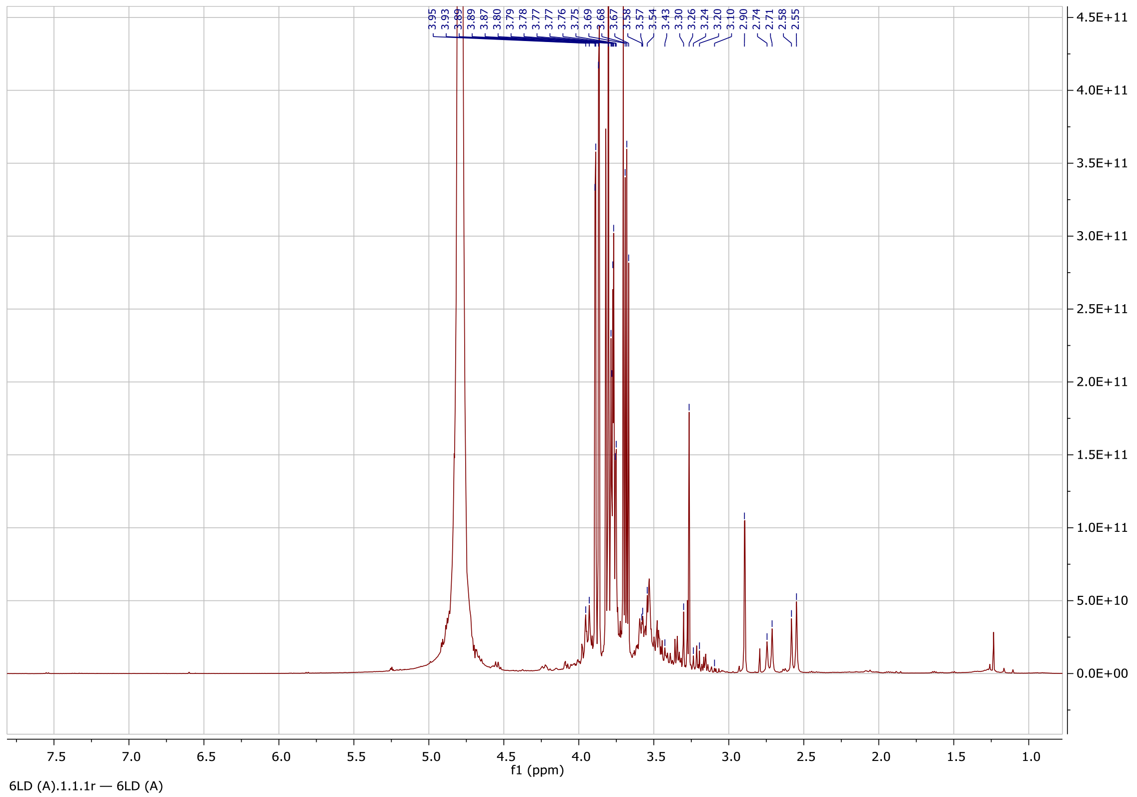

Supplementary Figure 12: ^{1}H NMR spectrum of *Lobophora dagamae* in D_2O

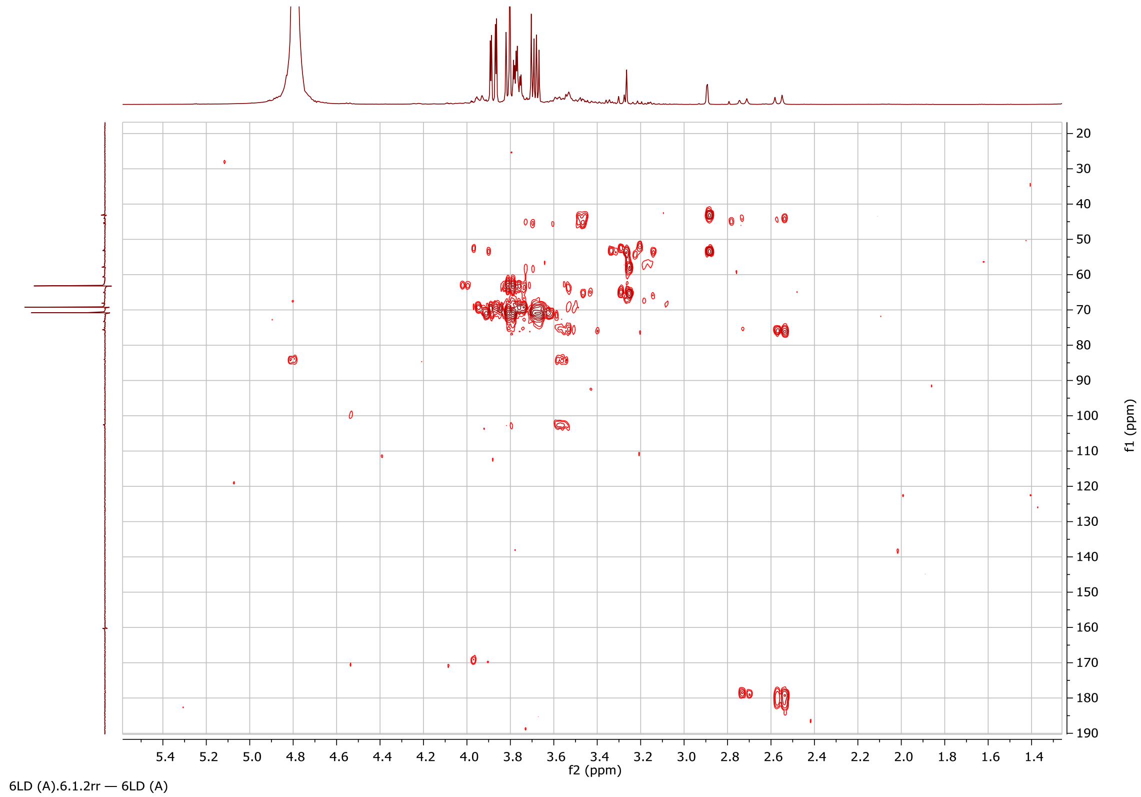

Supplementary Figure 13: HMBC spectrum of *Lobophora dagamae* in D_2O

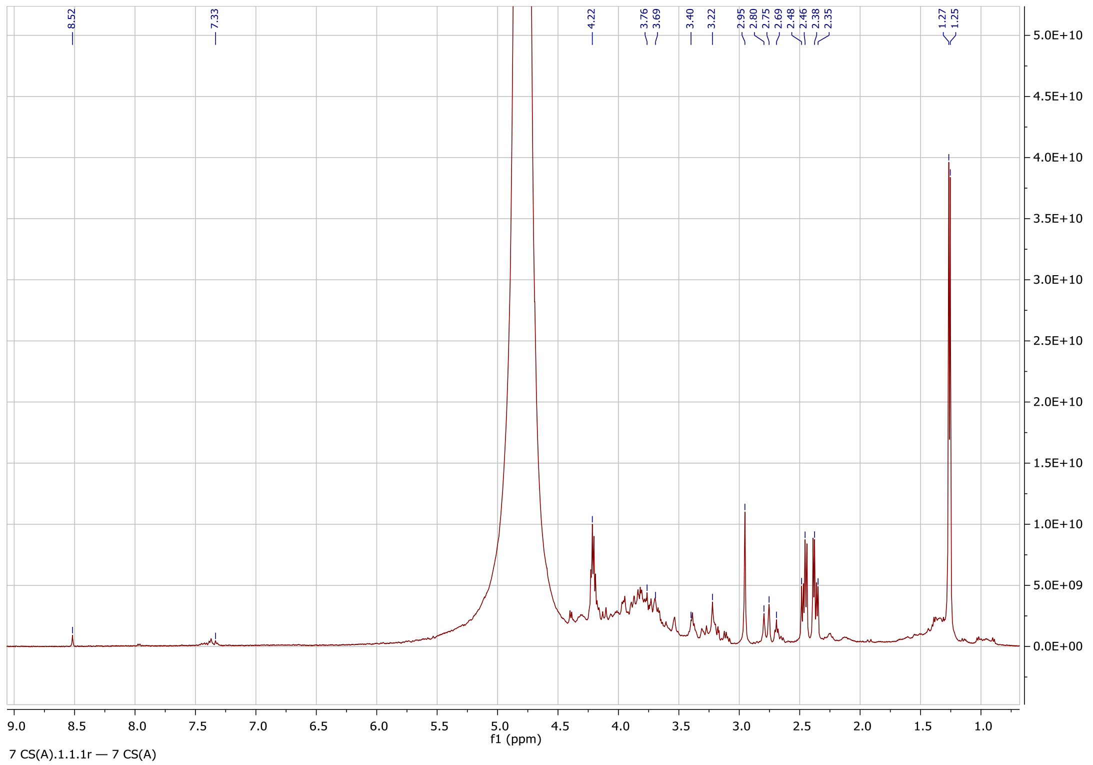

Supplementary Figure 14: ^{1}H NMR spectrum of *Colpomenia sinuosa* in D_2O

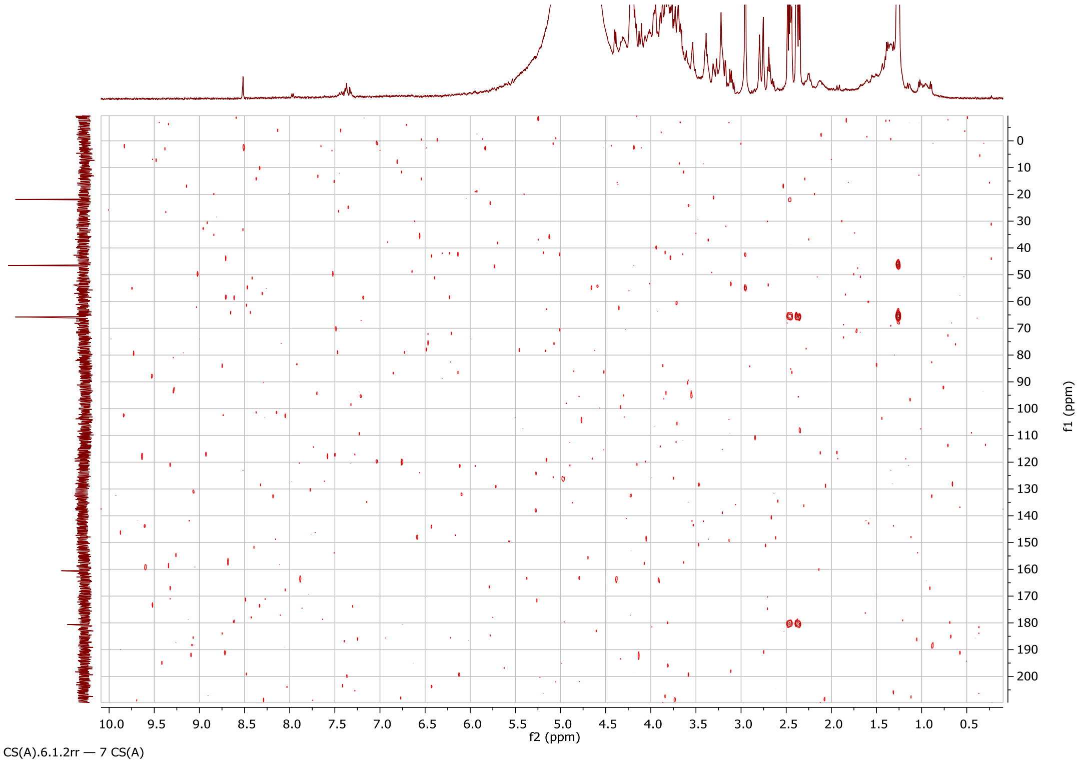

Supplementary Figure 15: HMBC spectrum of *Colpomenia sinuosa* in D_2O

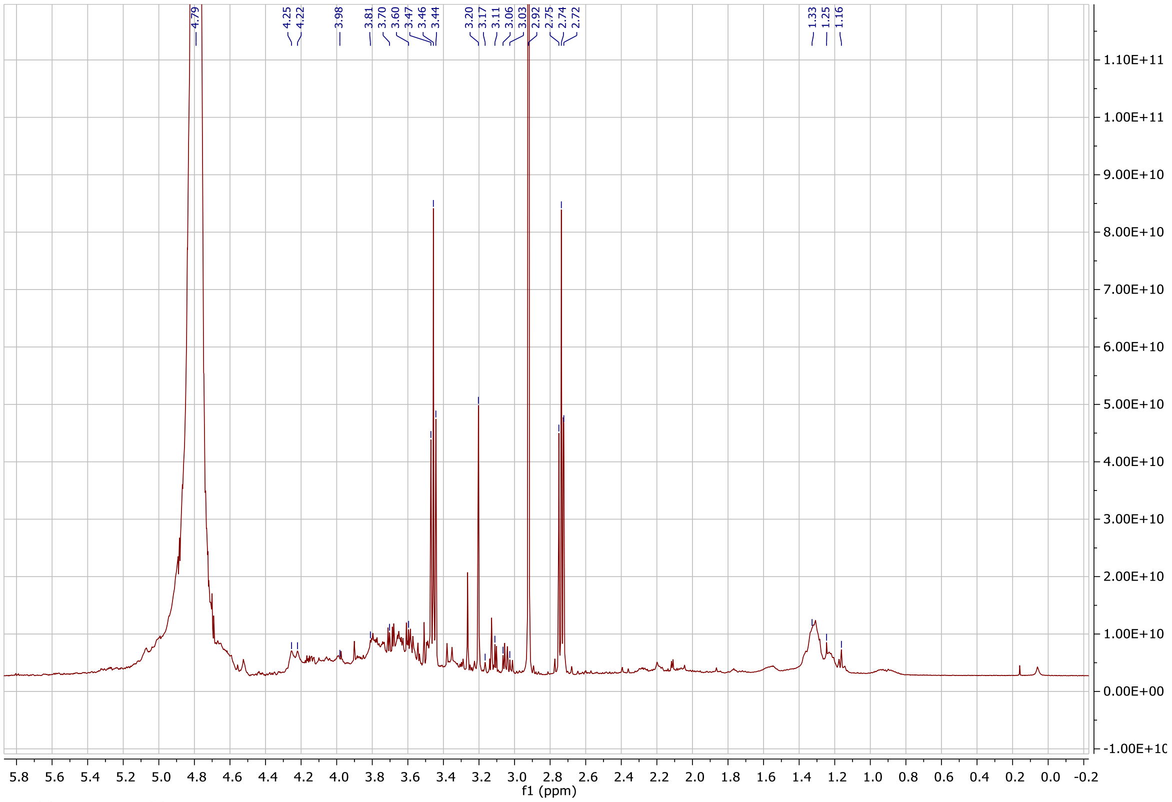

Supplementary Figure 16: ^{1}H NMR spectrum of *Ulva clathrata* in D_2O

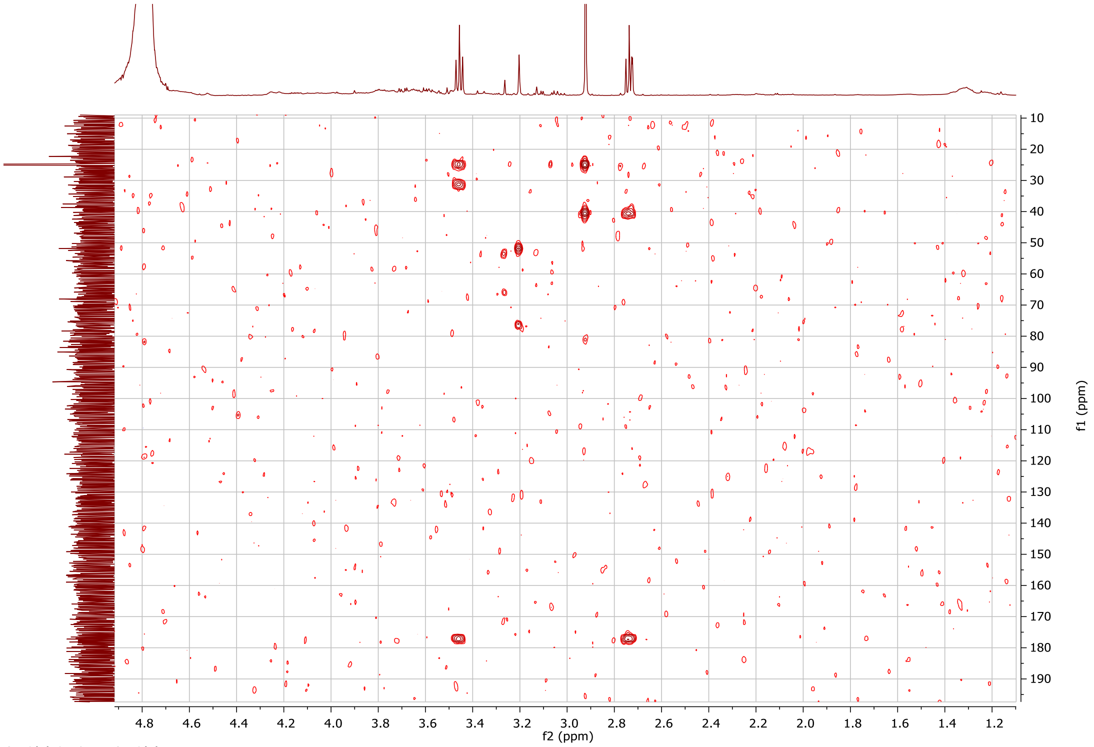

Supplementary Figure 17: HMBC spectrum of *Ulva clathrata* in D_2O

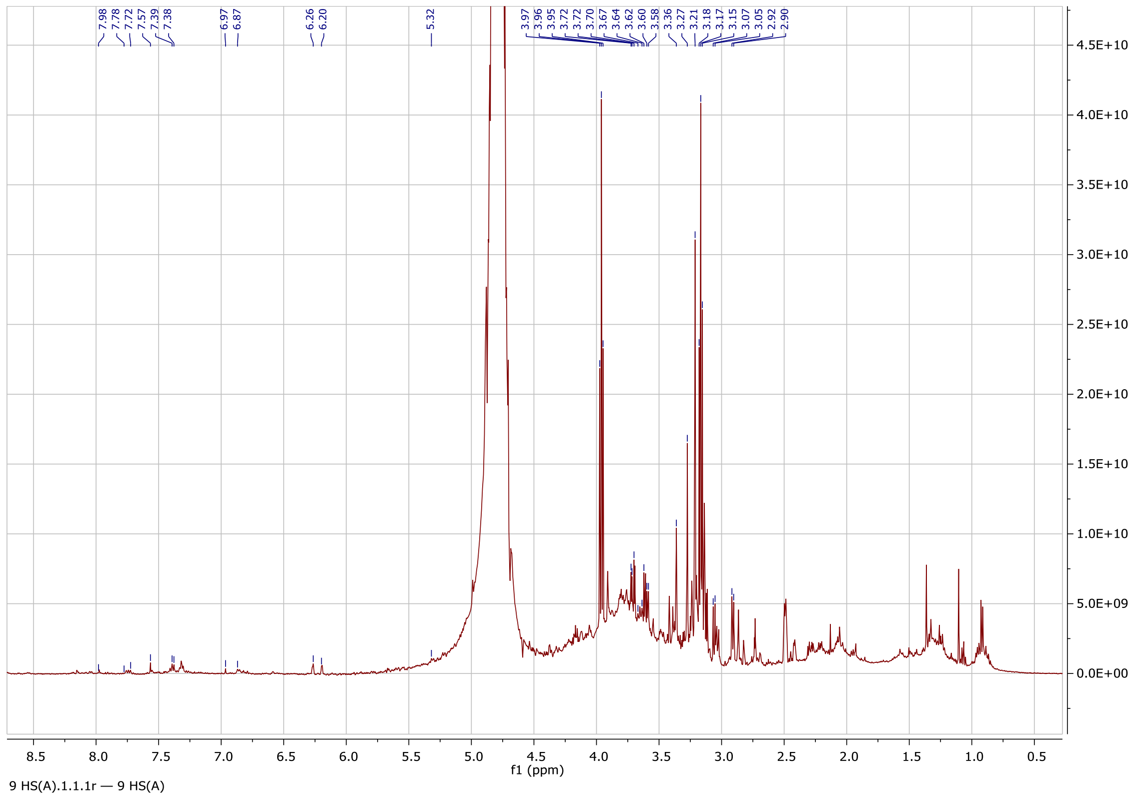

Supplementary Figure 18: ^{1}H NMR spectrum of *Halopteris scoparia* in D_2O

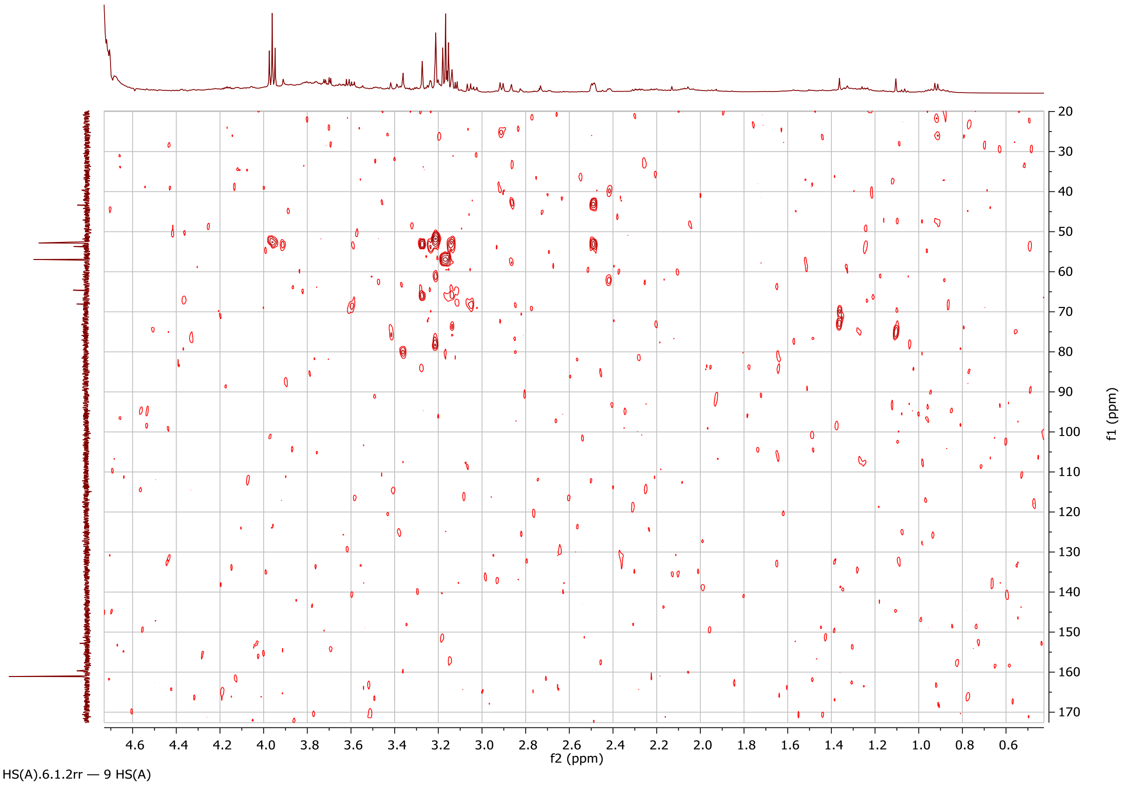

Supplementary Figure 19: HMBC spectrum of *Halopteris scoparia* in D_2O

frontiers

G. rugosa extract

d6-ABA

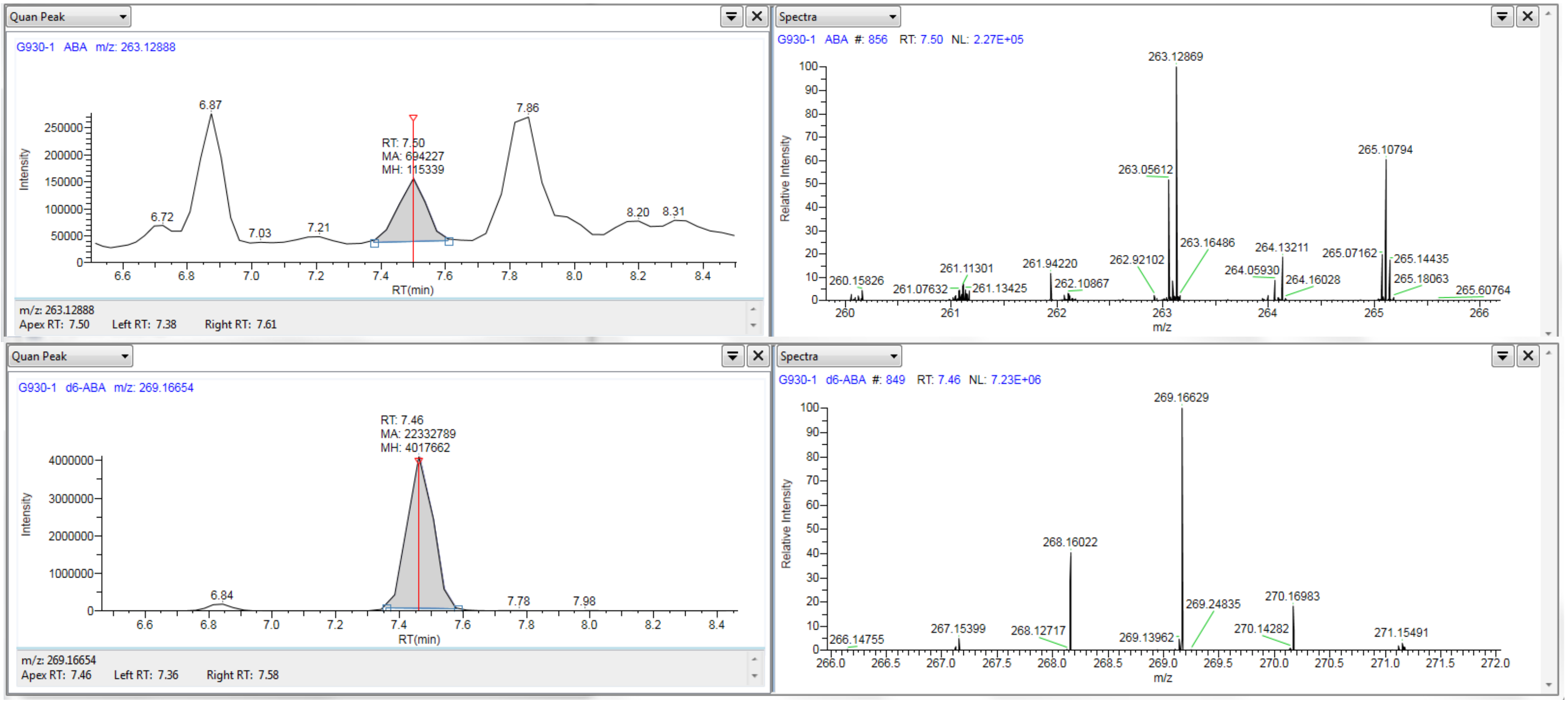

Supplementary Figure 20. ABA quantification. Reverse phase UHPLC chromatography (left) and Q Exactive Orbitrap Mass Spectrometer analysis (right) of the *G. rugosa* extract and the deuterium-labeled (d6) ABA. Note that the m/z ratio for the d6-ABA is 6 units higher because the internal standard is hexadeuteroabscisic acid.
